# Automated visualization of rule-based models

**DOI:** 10.1371/journal.pcbi.1005857

**Published:** 2017-11-13

**Authors:** John Arul Prakash Sekar, Jose-Juan Tapia, James R. Faeder

**Affiliations:** Department of Computational & Systems Biology, University of Pittsburgh, Pittsburgh, PA, United States of America; University of Virginia, UNITED STATES

## Abstract

Frameworks such as BioNetGen, Kappa and Simmune use “reaction rules” to specify biochemical interactions compactly, where each rule specifies a mechanism such as binding or phosphorylation and its structural requirements. Current rule-based models of signaling pathways have tens to hundreds of rules, and these numbers are expected to increase as more molecule types and pathways are added. Visual representations are critical for conveying rule-based models, but current approaches to show rules and interactions between rules scale poorly with model size. Also, inferring design motifs that emerge from biochemical interactions is an open problem, so current approaches to visualize model architecture rely on manual interpretation of the model. Here, we present three new visualization tools that constitute an automated visualization framework for rule-based models: (i) a *compact rule visualization* that efficiently displays each rule, (ii) the *atom-rule graph* that conveys regulatory interactions in the model as a bipartite network, and (iii) a tunable *compression pipeline* that incorporates expert knowledge and produces compact diagrams of model architecture when applied to the atom-rule graph. The compressed graphs convey network motifs and architectural features useful for understanding both small and large rule-based models, as we show by application to specific examples. Our tools also produce more readable diagrams than current approaches, as we show by comparing visualizations of 27 published models using standard graph metrics. We provide an implementation in the open source and freely available BioNetGen framework, but the underlying methods are general and can be applied to rule-based models from the Kappa and Simmune frameworks also. We expect that these tools will promote communication and analysis of rule-based models and their eventual integration into comprehensive whole-cell models.

## Author summary

Signaling in living cells is mediated through a complex network of chemical interactions. Current predictive models of signal pathways have hundreds of reaction rules that specify chemical interactions, and a comprehensive model of a stem cell or cancer cell would be expected to have many more. Visualizations of rules and their interactions are needed to navigate, organize, communicate and analyze large signaling models. In this work, we have developed: (i) a novel visualization for individual rules that compactly conveys what each rule does, (ii) a comprehensive visualization of a set of rules as a network of regulatory interactions called an atom-rule (AR) graph, and (iii) a set of procedures for compressing the AR graph into a pathway diagram that highlights underlying signaling motifs such as feedback and feed-forward loops. We show that these visualizations are compact and informative across models of widely varying sizes. The methods developed here not only improve the understandability of current models, but also establish principles for organizing the much larger models of the future.

This is a *PLOS Computational Biology* Methods paper.

## Introduction

Rule-based frameworks such as BioNetGen [1–3], Kappa [4–6] and Simmune [7,8] have been used to build detailed kinetic models of signaling pathways (e.g., FcεRI [9–11], TCR [12], EGFR [13,14], and p53 [15]). A rule-based model is composed of multiple “reaction rules”, where each rule specifies a reaction mechanism and its structural requirements, e.g., a phosphorylation rule would specify the set of binding interactions that bring the kinase into contact with substrate and the specific site on the substrate that is phosphorylated. Current models range in size from tens to hundreds of reaction rules, but these numbers are expected to increase as rule-based models are collectively organized in databases of kinetic interactions [10,12,14,16] and eventually integrated into whole cell models [17]. Large models, whether rule-based or otherwise, are difficult to understand or communicate without good visualization methods. Currently, the size of rule-based model that can be simulated far exceeds the size of model for which useful visualizations can be constructed automatically. In particular, we do not have visualizations that can present the regulatory interactions embedded in a model as a network diagram of signal flows. Also, other than using manual approaches, we do not have an effective approach to build compact pathway diagrams to communicate the model. Solving the automated diagramming problem is necessary to make the leap from opaque machine-readable model descriptions that can only be understood through manual annotation to transparent models that can be understood and explored by the wider community.

**Fig 1:**
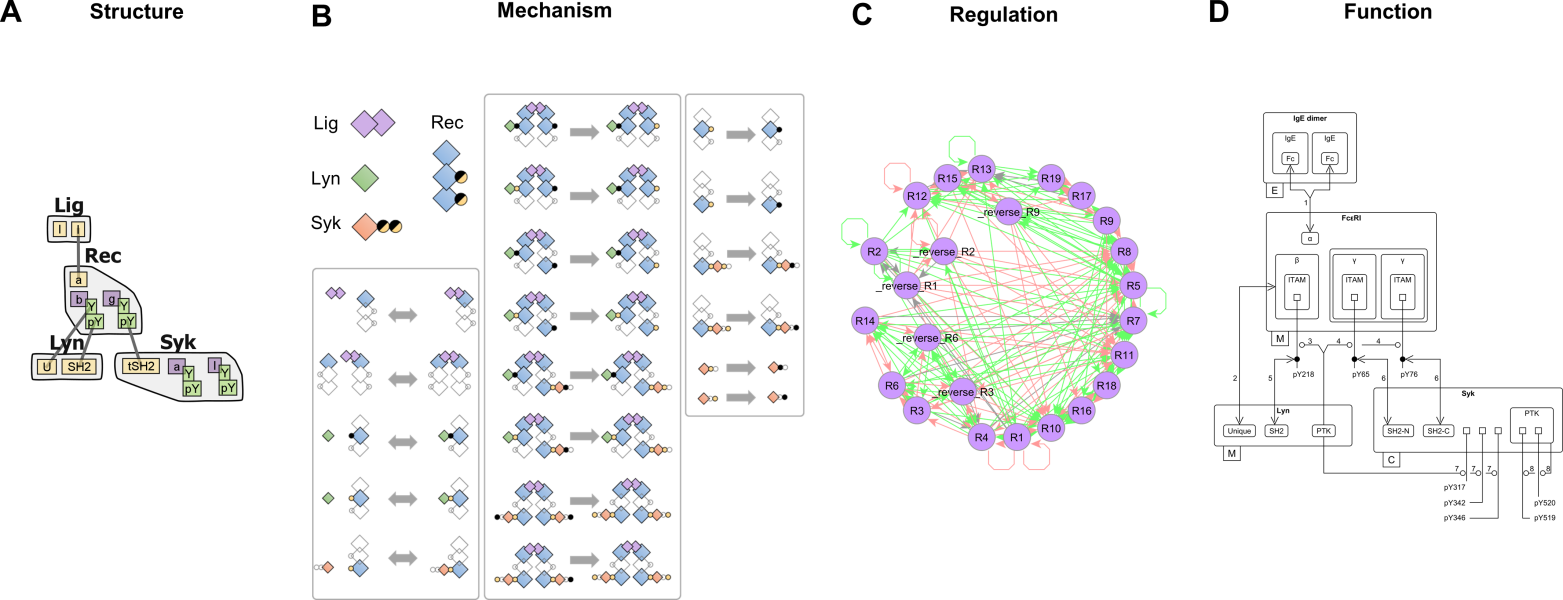
Visualization of an immunoreceptor signaling model [9]. (A) Contact map showing the types of molecules in the model (Lig, Rec, Lyn, Syk), their components, and the internal states (*Y*,*pY*) and bonds available to those components. (B) The 24 rules in the model visualized as reactant-to-product transformations, showing what structures need to be matched for each rule to be triggered. (C) Rule influence diagram showing interactions between pairs of rules, computed by explicitly comparing each pair of rules from panel B. (D) Extended Contact Map drawn by manually interpreting the model according to a defined set of conventions [23]. Each diagram in panels B-D raises specific complexity and usability issues (see main text).

Why is it challenging to visualize rule-based models? Tools that formally visualize the model tend to focus on a single type of information, such as what molecular structures are being modeled (contact map [6]), what rules have been defined on those structures (Simmune [8], Virtual Cell [18,19], BioUML [20]), and how various rules interact with each other (rule influence diagram [21], Kappa story [22]). To communicate the architecture of the model at a global level, these different types of information have to be integrated into a single diagram, but current approaches such as the Extended Contact Map (ECM) [23], the Systems Biology Graphical Notation: Entity Relationship Diagram (SBGN:ER) [24] and the Molecular Interaction Map (MIM) [25] rely on human interpretation, which decouples the diagram from the executable model. Methods to automate generation of diagrams include the Simmune Network Viewer [26], which uses an interactive approach to visualization, and the *rxncon* regulatory graph [27], which has a simplified representation of rule-based models that is more amenable for visualization than standard rules. In Fig 1, we apply a contact map, a conventional rule visualization approach, a rule influence diagram and an extended contact map to a previously published model of immunoreceptor signaling [9], and below, we discuss the issues raised by each type of information displayed in those diagrams. We also present more detailed comparisons to the remaining tools in Discussion.

The contact map (Fig 1A) conveys the structural composition of a model (e.g. in [28–30] and others) by showing what types of molecular structures are available to compose reaction rules [6]. This includes structured objects called molecules, components, states and bonds, which we explain in more detail in the Methods section.

Conventional rule visualizations (Fig 1B) show reaction rules as reactant to product transformations. The reactant side includes not just the structures that are to be modified in the rule, but also the structural requirements that need to be matched for the rule to be triggered. To determine the action of a rule, the reader has to compare reactants to products, which can be challenging for complex rules that have a number of structural requirements (e.g., rules in the center column of Fig 1B). Nevertheless, this is the standard approach to show rules (e.g., in [9,13,28] and others), whether using manually drawn diagrams such as Fig 1B or automated diagrams generated by various software (Simmune [8], Virtual Cell [18,19], BioUML [20]).

The rule influence diagram (Fig 1C) represents each rule with a single node and each computed interaction between rules as a directed edge [21,31]. Each rule interacts with other rules through shared structures, e.g., a binding rule that produces a kinase-bound configuration regulates a phosphorylation rule that requires the same configuration. However, it is difficult to understand regulatory interactions from just the rule influence diagram because it does not show structures interacting with rules. Also, even moderate-sized models produce unreadably dense diagrams such as Fig 1C, and the computation of influences is quadratic in the number of rules, which is limiting for large models. Both BioNetGen and Kappa frameworks can generate rule influence diagrams, with the Kappa version allowing for different levels of precision [31].

The extended contact map (Fig 1D) is an expert-curated diagram that highlights functional roles of various structures and mechanisms as well as emergent regulatory architectures such as feedbacks and cascades [23]. It uses standard diagramming conventions to convey function (e.g., round arrowhead to indicate phosphorylation), annotation to relate diagram to model (e.g., edge label 2 pointing to rule number 2), and secondary documentation to convey biological significance (e.g., an attached model guide that indexes and describes each rule). Each of these components is constructed manually, which is also true for related methods such as SBGN:ER [24] and MIM [25] (see Discussion). Several recent models make use of the ECM ([10,12,14,32] and others).

In this work, we introduce three new methods that together constitute a new visualization framework for rule-based models. First, we introduce a novel *compact rule visualization*, which is more concise than conventional representations of rules and does not require visual comparison to convey the action of the rule. Second, we develop the *atom-rule (AR) graph* for showing regulatory interactions that can be efficiently derived from rules without pairwise comparisons. The bipartite AR graph displays a global view of how rules interact through the structures present in a model. Finally, because the raw AR graph is too dense for many applications, we present an AR graph compression pipeline that integrates expert knowledge and generates more readable diagrams. These methods are compatible with rules from the three widely-used frameworks of BioNetGen [1–3], Kappa [4–6] and Simmune [7,8] and also with the proposed interchange format SBML-multi [33]. We have provided an implementation in BioNetGen 2.2 [3], which is already available to users and to frameworks that incorporate BioNetGen, such as PySB [34] and Virtual Cell [18,19].

The remainder of the paper is organized as follows. In Methods, we briefly describe the new visualization methods and apply them to simple examples. In Results, we apply the methods to larger and more complex models, including a test set of 27 rule-based models from the literature. We use standard measures of graph readability to show that our methods produce more readable diagrams than current alternatives. In Discussion, we present additional comparisons with existing tools and discuss the potential benefits of the new tools for analysis of rule-based models.

## Methods

The frameworks of BioNetGen [1–3], Kappa [4–6] and Simmune [7,8] share similar rule-based representations for which several formal treatments have been presented in the literature (BioNetGen [35–37], Kappa [4,5,22]). The visualization tools developed in this work have been implemented in BioNetGen, but operate on features of rule-based modeling common to all three frameworks. We recommend Chylek *et al*. [38] for a recent review of rule-based modeling, Sekar *et al*. [39] for a BioNetGen tutorial, and Hogg *et al*. (Supplement) [36] for a description of the BioNetGen formalism. In this section, we use a simple rule-based model to introduce reaction rule syntax and semantics, then demonstrate our new visualization approaches, namely compact rule visualizations and atom-rule graphs. S1 Appendix provides a more detailed theoretical foundation as well as specifications for algorithms and rendering conventions. S2 Appendix provides a step-by-step tutorial for applying methods to a complex signaling model from Suderman and Deeds [40].

### Reaction rules

**Fig 2:**
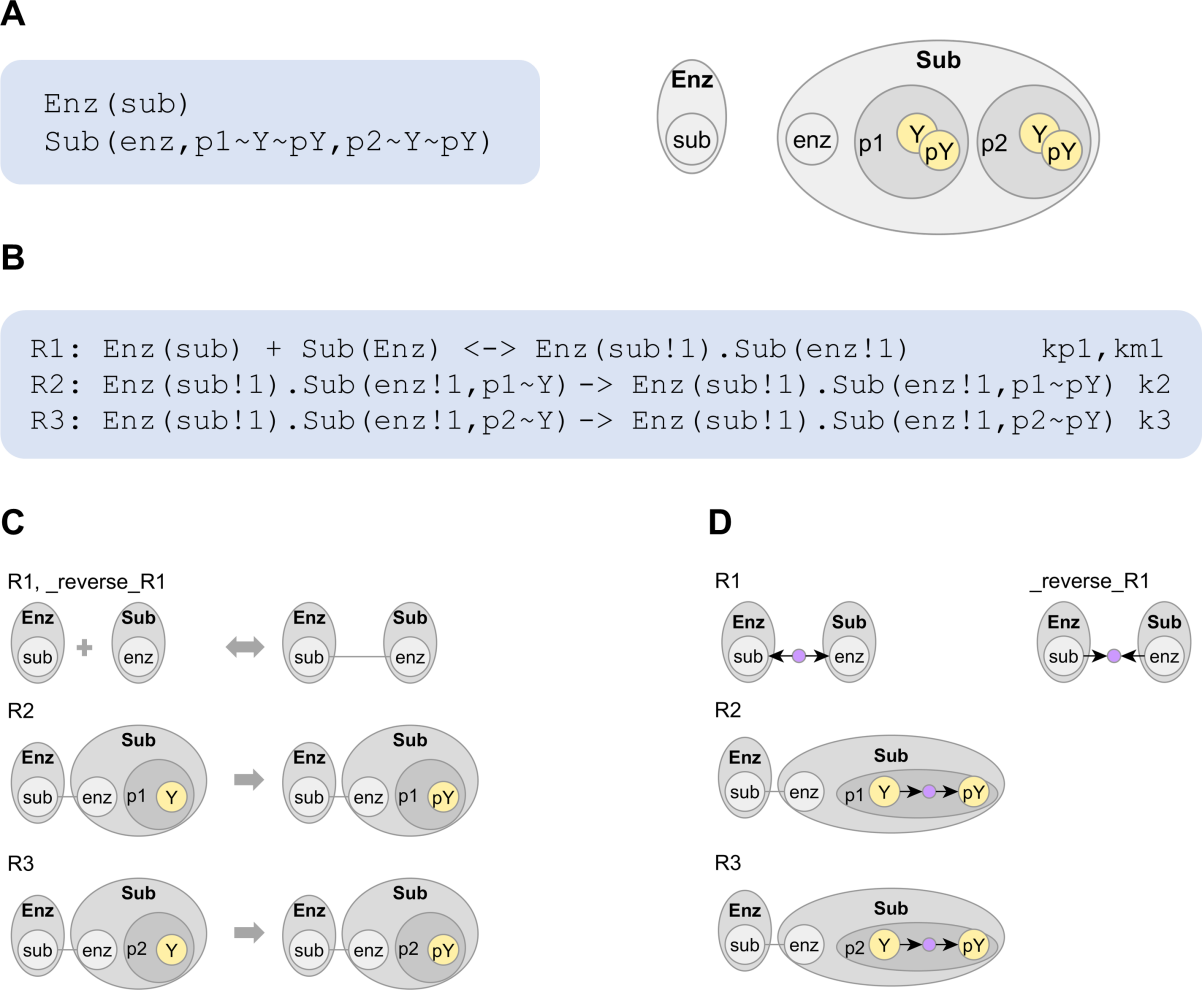
Visualizing rules of an enzyme-substrate system. (A) Structured molecule types Enz and Sub, their respective components (enclosed in ‘()’) and their available internal states (prefixed by ~), shown in BioNetGen syntax and graphic. (B) Reaction rules specifying the adding and removing of an enzyme-substrate bond (binding partners tagged by !1) and phosphorylation of components *p1* and *p2*, shown in BioNetGen syntax. The reactants and products of a reaction rule are called patterns. (C) Conventional rule visualization by drawing reactant and product patterns separately. (D) Compact rule visualization displays operations (purple nodes) that transform the reactant patterns in each rule. On operation nodes, outward edges indicate that a new structure is produced (bond in R1, state *pY* in R2 and R3) and inward edges indicate that a structure is consumed (bond in _reverse_R1, state *Y* in R2 and R3). To see operation nodes with their labels, see S1 Fig.

In a rule-based model, **molecules** are structured objects composed of **components**. Fig 2A shows the BioNetGen language (BNGL) specification of molecules Enz and Sub representing enzyme and substrate respectively, along with corresponding visualizations. Enz has component *sub* and Sub has components *enz*, *p1* and *p2*. By convention, a component with a binding function is named after the molecule that it binds. So, *sub* on enzyme and *enz* on substrate represent binding sites for substrate and enzyme respectively. Components *p1* and *p2* represent phosphorylation sites.

A component may have one or more modifications available to it, called **internal states**. For example, components *p1* and *p2* may be in the unphosphorylated state *Y* or phosphorylated state *pY*. **Bonds** can occur between pairs of components. Here, component *sub* on an Enz molecule can bind component *enz* on a Sub molecule to form an enzyme-substrate complex. **Patterns,** which are constructed from molecules, components, internal states and bonds, specify the reactants and products of a **reaction rule**. In Fig 2B, we show the BNGL specification of a simple enzyme-substrate system. Each rule requires a rate constant, with reversible rules, such as R1, requiring rate constants for both forward and reverse directions. In Fig 2C, we visualize the rules using a conventional approach.

Each reaction rule explicitly encodes model assumptions about a reaction mechanism. Structural features specified on the reactant side and modified on the product side constitute the **reaction center.** In rule R1 and its reverse, the *sub-enz* bond is formed in the forward direction and removed in the reverse direction, which indicates that R1 models reversible enzyme-substrate binding. In rule R2, the unphosphorylated state of *p1* is transformed to the phosphorylated state, which indicates that R2 models phosphorylation of component *p1*. Analogously, rule R3 models phosphorylation of component *p2*.

Features that remain the same on both sides of a rule constitute **reaction context**, which describes the local conditions necessary for the mechanism to occur. In rules R2 and R3, the *sub-enz* bond is present on both sides of the rule, which indicates that the respective phosphorylation mechanisms require the enzyme-substrate binding interaction. Features omitted on both sides of the rule are assumed not to affect the reaction mechanism. Components *p1* and *p2* are omitted in rule R1 and its reverse, which specifies that binding and unbinding mechanisms are independent of *p1* and *p2*. Similarly, rules R2 and R3 specify that phosphorylation at *p1* is independent of *p2* and *vice versa*.

### Conventional rule visualization

The **site graph** is a nested graph used to represent patterns [22], such as the reactants and products in Fig 2C. In this work, we use site graph to refer to the visualization scheme where nodes representing molecules, components and internal states are nested hierarchically and bonds are shown as edges between components.

In **conventional rule visualization**, as shown in Fig 2C, each reactant and product pattern is drawn separately as a site graph. To distinguish reaction center and reaction context, e.g., to identify that rule rule R2 transforms the internal state of *p1* and requires the *sub-enz* bond, the viewer has to visually compare the graphs from each side of the rule. This imposes a high mental load for complex rules, especially when a large amount of context obscures a much smaller reaction center.

### Compact rule visualization

In this work, we introduce **compact rule visualization** (Fig 2D), which does not require visual graph comparison and avoids drawing reaction context twice. We describe its derivation in S1 Appendix. Briefly, we identify and merge structures common to both sides of the rule, then use special nodes called **graph operation nodes** to represent the modifications performed. The directions of edges on the graph operation node indicate whether a structure is consumed or produced by that operation. In Fig 2D, each rule is shown with the respective operation node, namely AddBond (R1), DeleteBond (_reverse_R1), and ChangeState (R2, R3) respectively. BioNetGen also supports creating and deleting molecules (AddMol, DeleteMol) and multiple operations per rule (S1 Fig).

To interpret compact rule visualization, the viewer looks for graph operation nodes, which are visually distinguishable from molecule, component and internal state nodes. The structures adjacent to the graph operation nodes constitute the reaction center, whereas the remaining structures constitute reaction context.

### Atom-rule graphs

In this work, we introduce atoms and atom-rule graphs, which enable visualizing the regulatory architecture represented by a set of reaction rules.

**Fig 3:**
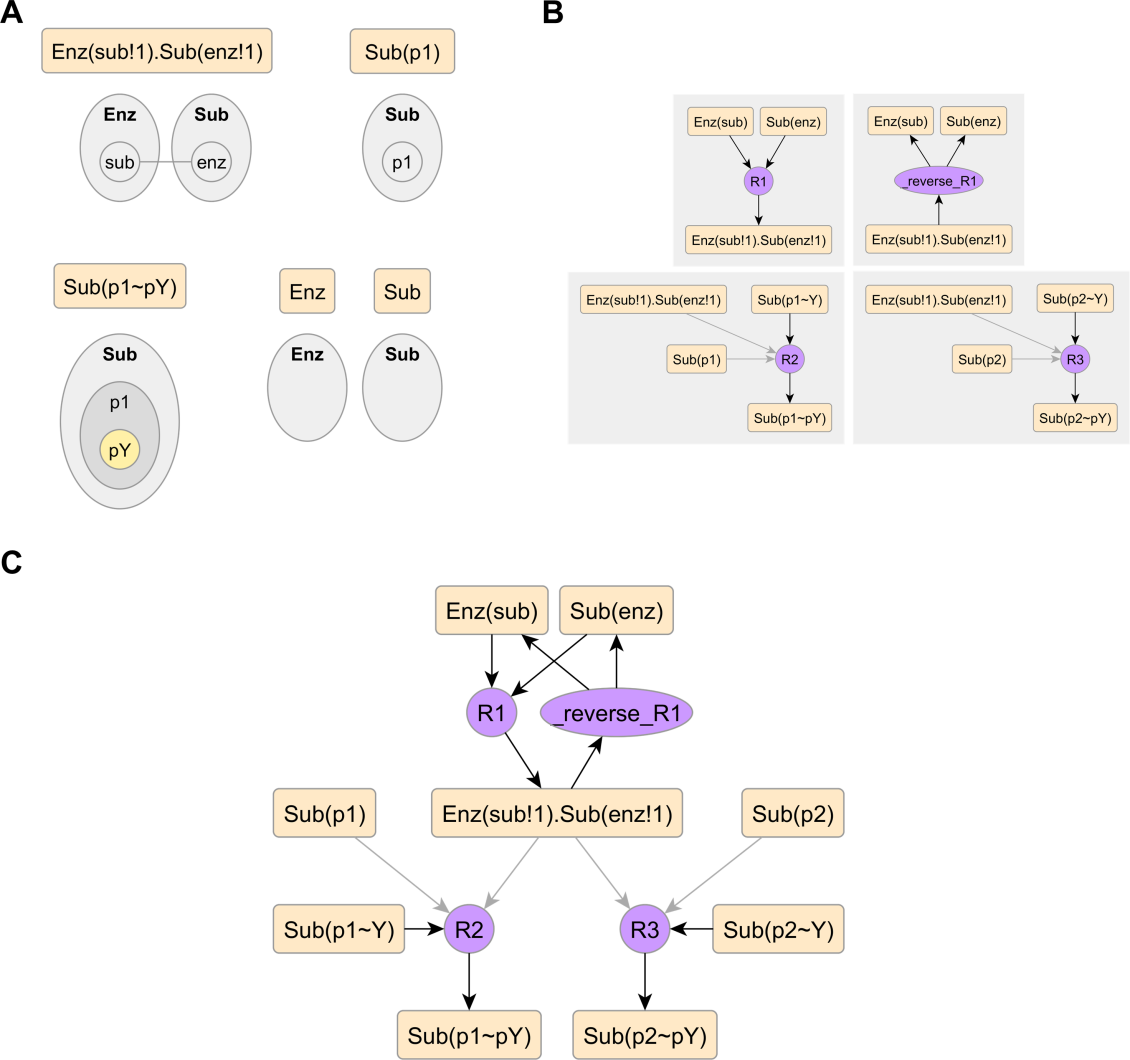
Atom-rule (AR) graphs. (A) Atoms, which are elementary structural features, shown in BioNetGen syntax and graphic. (B) Atom-rule graphs derived from the individual rules in Fig 2B. (C) The full atom-rule graph of the model, merged from individual graphs in panel B. AR graphs have three edge types: reactant (dark color, pointed towards rule), product (dark color, pointed away from rule), and context (light color, pointed towards rule), which indicate whether an instance of an atom is present in the rule’s reaction center (reactant/product) or reaction context.

**Atoms** are elementary structural features found in patterns. In Fig 3A, using BioNetGen syntax as well as site graph visuals, we show instances of various types of atoms present in the product pattern of rule R2. They include:

- molecule atoms, such as Enz and Sub,
- free binding site atoms, such as Sub(p1)
- internal state atoms, such as Sub(p1~pY)
- bond atoms, such as Enz(sub!1).Sub(enz!1)

The **Atom-Rule (AR) graph** indicates the relationship of a rule with various atoms, which can be reactant, product and/or context. We describe its derivation in detail in S1 Appendix. Briefly, a reactant or product edge is drawn if an instance of the atom is present in the reaction center, on the left or right side of the rule respectively. A context edge is drawn if an instance is present in the reaction context. In Fig 3D, we show AR graphs derived from the rules in Fig 2C, with atomic node labels in BioNetGen syntax. For convenience, the molecule atoms are omitted if there are no molecules added or deleted in the rule.

To interpret the AR graph, one views each atom as a class of actionable sites present in the model. For example, Sub(p1~Y) represents the class of unphosphorylated states on *p1* components that can potentially be acted upon by phosphorylation mechanisms. Then, one interprets each edge as an interaction between a mechanism and a class of sites. A reactant or product edge respectively indicates that a mechanism has a consumption or production effect on that particular class of sites. A context edge indicates that the mechanism requires that particular class of sites as a local condition. For example, from the AR graph of rule R2 in Fig 3B, we infer that R2 consumes unphosphorylated *p1*, produces phosphorylated *p1*, and requires that *p1* be unbound and that enzyme be bound to substrate.

The **model AR graph**, as in Fig 3C, is a bipartite graph between rules and atoms that is constructed by merging AR graphs of individual rules. Paths on the model AR graph that alternate between rules and atoms represent signal flows. A particular set of rules will always produce the same AR graph, which is a complete representation of signal flow in that rule set between atoms and rules.

### Compressing AR graphs

**Fig 4:**
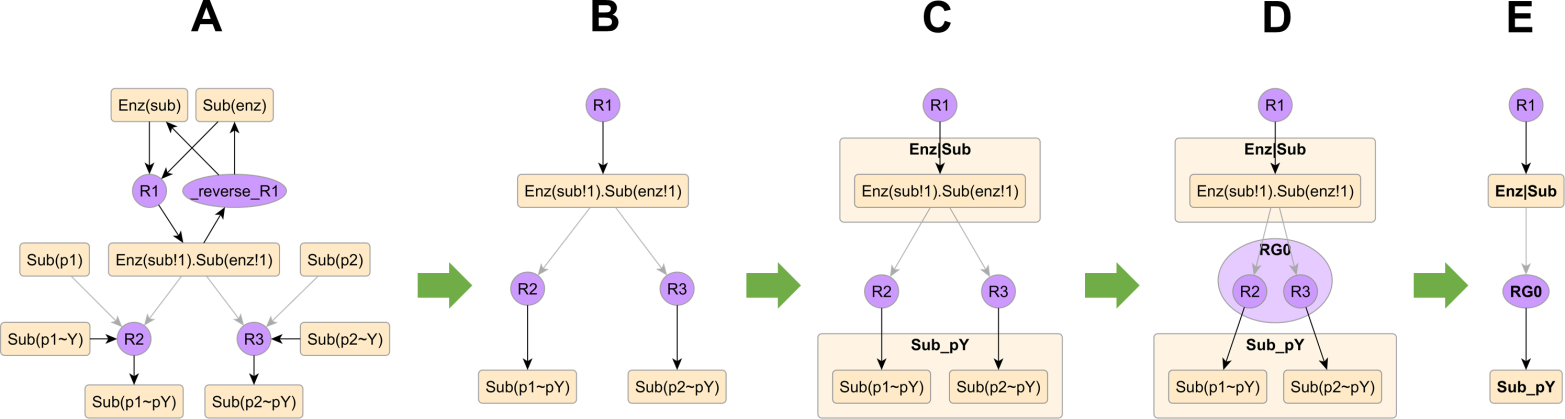
Compressing AR graphs. (A) The full model AR graph from Fig 3C. (B) Removing atoms and rules with low priority, such as free binding sites, unphosphorylated states and the unbinding rule. (C) Grouping structurally similar atoms, e.g., Sub_pY = phosphorylated states of *p1* and *p2*, Enz|Sub = bond linking Enz and Sub. (D) Grouping rules with similar edge signatures, e.g., rules R2 and R3 both have a context edge from Enz|Sub and a product edge into Sub_pY. (E) Each group is merged into a single node that combines all incident edges. Panels B-C are semi-automated, whereas panels D-E are fully automated.

To build compact pathway diagrams that convey function, we provide a pipeline for reducing the complexity of the model AR graph (Fig 4A) while preserving relevant regulatory features. Briefly, it involves:

1. Removing low-priority atoms and rules (Fig 4B).
2. Sorting atoms into groups (Fig 4C).
3. Sorting rules into groups (Fig 4D).
4. Merging groups of nodes and their incident edges (Fig 4E).

The output of this pipeline is the **compressed model AR graph.**

To decide which atoms and rules to remove (Step 1) as well as which atoms belong together as groups (Step 2), we take a semi-automated approach. An automated heuristic grounded in commonly encountered biological scenarios makes a first pass through the full AR graph and outputs a template file containing the choices made by the heuristic. To account for nuances of individual systems, the user can edit this template to make alternate choices and import it back into the visualization tool (see tutorial in S2 Appendix for a demonstration). Following this, an automated procedure examines each rule on the graph, the edges incident on the rule and the atom groups adjacent to the rule, then groups rules that share the same edge signature (Step 3). Currently, we support two types of edge signature: *strict*, which examines all three edge types, and *permissive*, which examines only reactant and product edges. Finally, an automated procedure replaces each group of nodes with a single representative node (Step 4). Edges incident on individual nodes are merged onto the representative node. A particular set of pipeline inputs (edge signature, template) will generate the same compressed AR graph, but these inputs can be tuned to produce different compressed AR graphs.

Each step in the pipeline has a specific interpretation. Atoms and rules that are removed represent structures and mechanisms with low functional priority, which are typically free binding sites, unphosphorylated states, unbinding rules and dephosphorylation rules. Atom groups represent functional categories of biological structures, e.g., the set of phosphorylation sites on a receptor. Rule groups represent categories of similarly acting mechanisms, e.g., phosphorylation mechanisms active at a particular group of sites. Merging groups is equivalent to reducing the resolution of the graph from individual sites and processes to broad categories of those elements. Permissive grouping also introduces a weaker semantic for the context edge on the compressed graph: a merged group node with a context edge implies that at least one of its members prior to merging had the same context edge.

### Implementation

An implementation of the methods described here is freely available as part of the open source BioNetGen distribution at http://bionetgen.org. A typical procedure involves calling a “visualize()” method from the BioNetGen model file with arguments for user input as well as a template file with edits, if applicable. The default template file can also be automatically generated as a text file. The typical visualization output is a file in GML format (graph modeling language) [41,42] encoding nodes, node labels, edges, edge directions and style attributes of nodes and edges such as color and shape. To lay out the graph, i.e., assign specific coordinates to nodes, we recommend using a third party application such as the yEd graph editor (http://yworks.com/yed), which was also used for the graphs shown in this paper. The tutorial in S2 Appendix provides a detailed walkthrough of the visualization tools using the model from Suderman and Deeds [40] as an example.

### Graph complexity analysis

We compiled a list of 27 rule-based models from the literature, which we list in S1 Table and attach in S1 Dataset. The models had 2239 rules in total, with the number of rules per model ranging from 6 to 625. We applied to these models a suite of nine visualization tools: contact map, conventional rule visualization, compact rule visualization, Simmune Network Viewer, rule influence diagram and atom-rule graphs at various steps in the complexity reduction pipeline: full model AR graph, AR graph with background removed, AR graph compressed using a strict edge signature, and AR graph compressed using a permissive edge signature. The compression pipeline was applied automatically by making default choices for prioritizing and grouping nodes. On the output graphs, we computed number of nodes *(n)* and number of edges per node *(e/n)*, counting hierarchical relationships between nodes also as edges. We present these statistics in the Results section.

### Computational complexity analysis

Pseudocode for the algorithms underlying the tools as well as a detailed accounting of computational costs is available in S1 Appendix. Briefly, for compact rule visualization, the rate-limiting step is building a correspondence map between left and right sides of the rule. Given a maximum finite rule size, the cost can be considered as *O(1)* per rule. Examining the rule with the correspondence map to synthesize the AR graph is also *O(1)* per rule. Merging AR graphs of individual rules, grouping rules and merging groups are all *O(n)*, where *n* is the number of rules.

## Results

### Visualizing interactions of reaction rules

**Fig 5:**
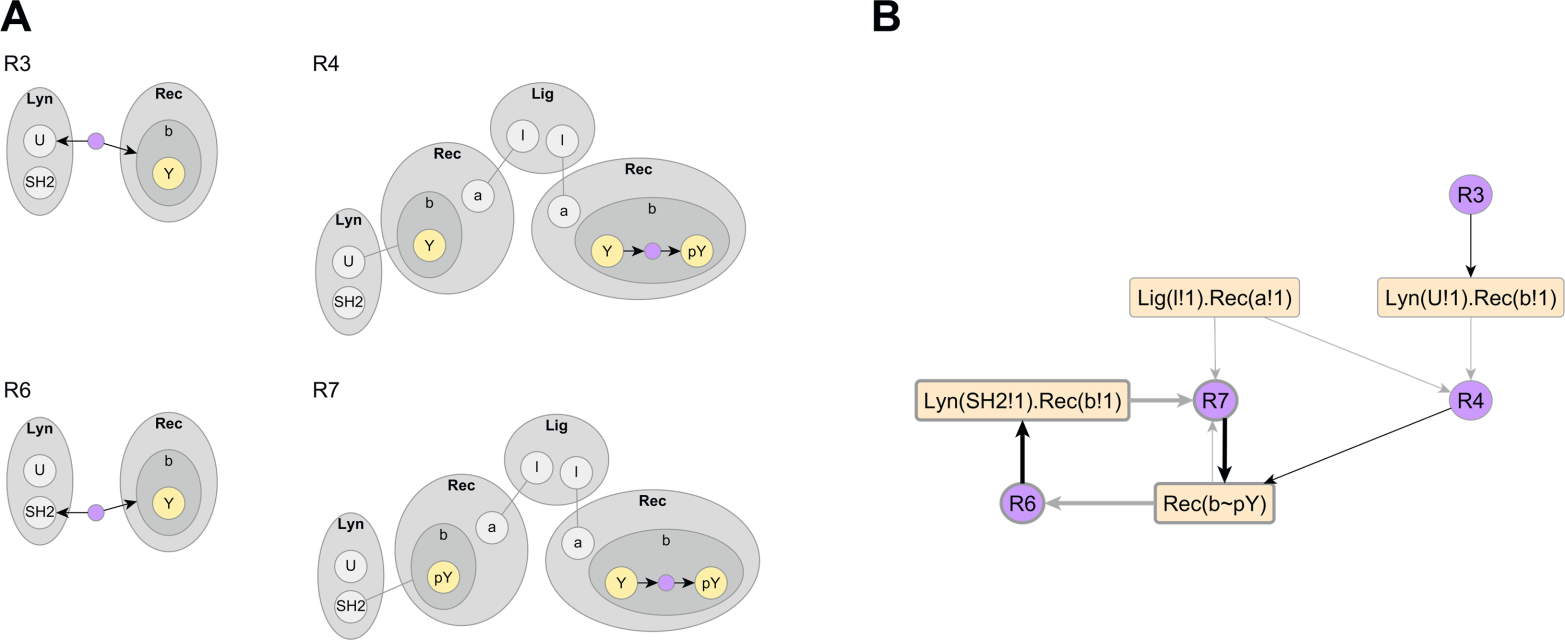
Visualizing Lyn-FcεRI interactions. (A) Lyn binds β domain of receptor that is unphosphorylated (constitutive binding–rule R3) or phosphorylated (activated binding–rule R6) via U or SH2 domains respectively. Recruited Lyn *trans-*phosphorylates β domain on the ligand-crosslinked receptor dimer (constitutively recruited Lyn–rule R4, actively recruited Lyn–rule R7). (B) The compressed atom-rule graph reveals a positive feedback loop between activated Lyn recruitment and receptor phosphorylation (highlighted with bold lines).

Visualizing individual rules promotes understanding the structural and kinetic assumptions encoded in a model. Unlike conventional rule diagrams, which require visually comparing reactant and product sides of a rule, *compact rule visualization* explicitly indicates which modification is performed on which set of structures. Specifically, it allows us to distinguish *reaction center*, the site of action of a rule, from *reaction context*, the structural requirements that need to be matched for the rule to fire. In Fig 5A, we show compact rule visualizations of four reaction rules from the immunoreceptor signaling model of Fig 1. Rules R3 and R6 have AddBond operations and represent two distinct binding modes of Lyn kinase to the β domain of FcεRI receptor. In R3, the U domain of Lyn binds the unphosphorylated β domain (constitutive binding), whereas in R6, the SH2 domain of Lyn binds the phosphorylated β domain (activated binding). Rules R4 and R7 have ChangeState operations and represent phosphorylation of the β domain in receptor dimers, with the active kinase being Lyn recruited through constitutive and activated modes respectively.

To understand a model, it is important to know how rules interact with each other and whether they form common motifs such as feedback or feedforward loops. For example, the rules in Fig 5A constitute a positive feedback loop: phosphorylation of β domain (R4, R7) activates Lyn binding (R6), which in turn promotes β phosphorylation (R7), but this is not obvious from conventional and compact rule visualizations. Current methods identify regulatory interactions between pairs of rules through graph comparison [21], simulation [6,22], or manual interpretation [23]. In contrast, the *atom-rule graph*, which is a bipartite graph showing regulatory interactions between rules and elementary structural features called *atoms* (see Methods), is constructed efficiently by examining each rule’s reaction center and reaction context. In Fig 5B, we show an AR graph constructed from rules R3, R4, R6 and R7, and the feedback loop is visible as a path on this graph.

### Tuning display of regulatory complexity

**Fig 6:**
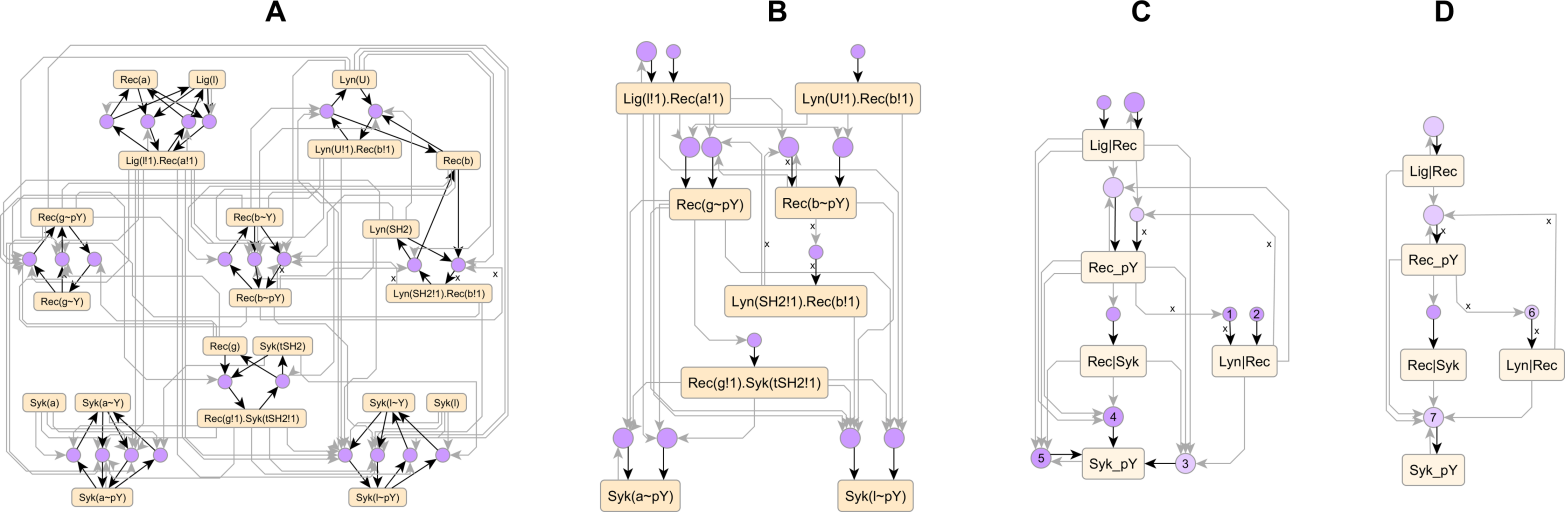
Atom-rule graphs of the immunoreceptor signaling model of Fig 1 at various levels of compression. (A) Full AR graph. (B) AR graph with low priority nodes removed. (C,D) Compressed AR graph following grouping and merging of nodes with a strict edge signature (C) or a permissive edge signature (D). For uncompressed versions of panels C-D, see S2B–S2C Fig.

The *model AR graph* for the full immunoreceptor model (Fig 6A) is a complete representation of signal flow in the model, encompassing all 24 rules. The compression pipeline (described in Methods) extracts the essential features of signal flow from the model AR graph and displays them as a compact pathway diagram. The steps of the pipeline, which we apply to the model AR graph in Fig 6A, include:

1. Removing atoms and rules with low priority,
2. Grouping structurally similar atoms,
3. Grouping rules with similar neighboring atoms,
4. Merging groups of atoms and rules.

In Step 1, we remove unphosphorylated states, dissociation rules and dephosphorylation rules from Fig 6A, producing the graph in Fig 6B. In Step 2, we group bonds that link the same molecules (Lig|Rec, Lyn|Rec, Rec|Syk) and phosphorylation sites on molecules (Rec_pY, Syk_pY), producing the atom groups shown in S2A Fig. In Step 3, grouping rules that share similar reaction centers and contexts produces the rule groups shown in S2B Fig, whereas dropping the context similarity requirement produces the more inclusive rule groups shown in S2C Fig. In Step 4, merging groups shown in S2B and S2C Fig produces the *compressed AR graphs* in Fig 6C and 6D respectively.

Unlike the full AR graph, the compressed graphs are compact and easier to understand. It is also easier to trace specific signal flows on the compressed graphs, such as the feedback between Lyn-receptor binding and receptor phosphorylation (edges marked *x* in Fig 6A–6D). Under default settings, the whole pipeline is automated, but the resolution of the compressed graphs and the quality of the output diagram can be tuned by providing user input, which includes customizing the heuristics for Steps 1 & 2 and choosing the grouping strategy for Step 3. The strict grouping used in Fig 6C resolves three variants of Syk phosphorylation under various contexts (nodes 1–3) and constitutive and phospho-activated Lyn|Rec binding modes (nodes 4–5), whereas the permissive grouping in Fig 6D merges variants of the same process and represents them with a single node (nodes 6,7). A specific set of pipeline inputs reproducibly generates the same compressed graph from the model and serves as diagram documentation.

### Visualizing reaction rule libraries

**Fig 7:**
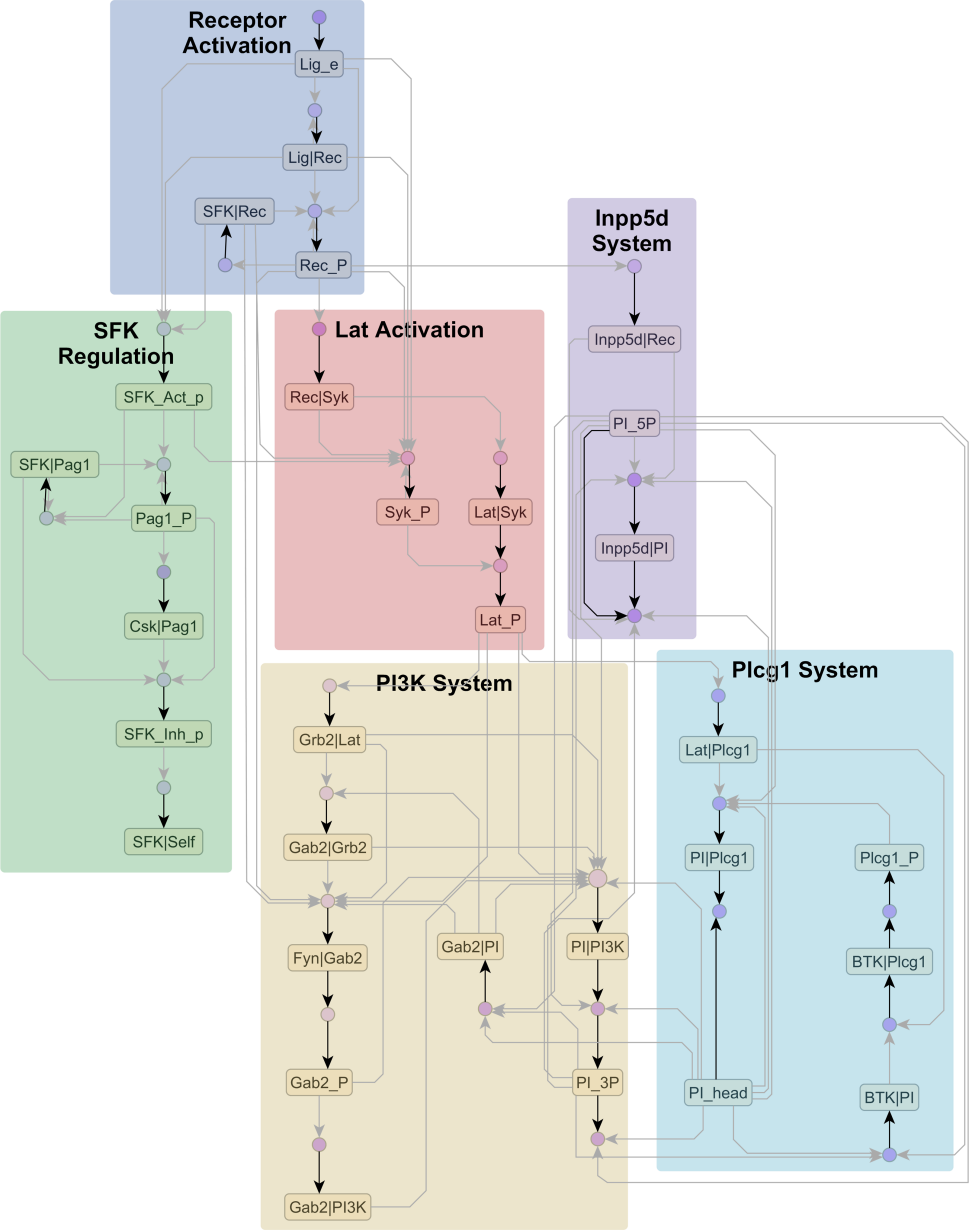
FcεRI library of rules. Compressed AR graph (63 nodes, 112 edges) generated from the FcεRI model of Chylek et al. [10] with 178 rules. The uncompressed graph has 305 nodes and 1076 edges. The model elements can be roughly classified into six subsystems shown above. The files needed to reproduce this diagram are provided in S1 Dataset.

**Fig 8:**
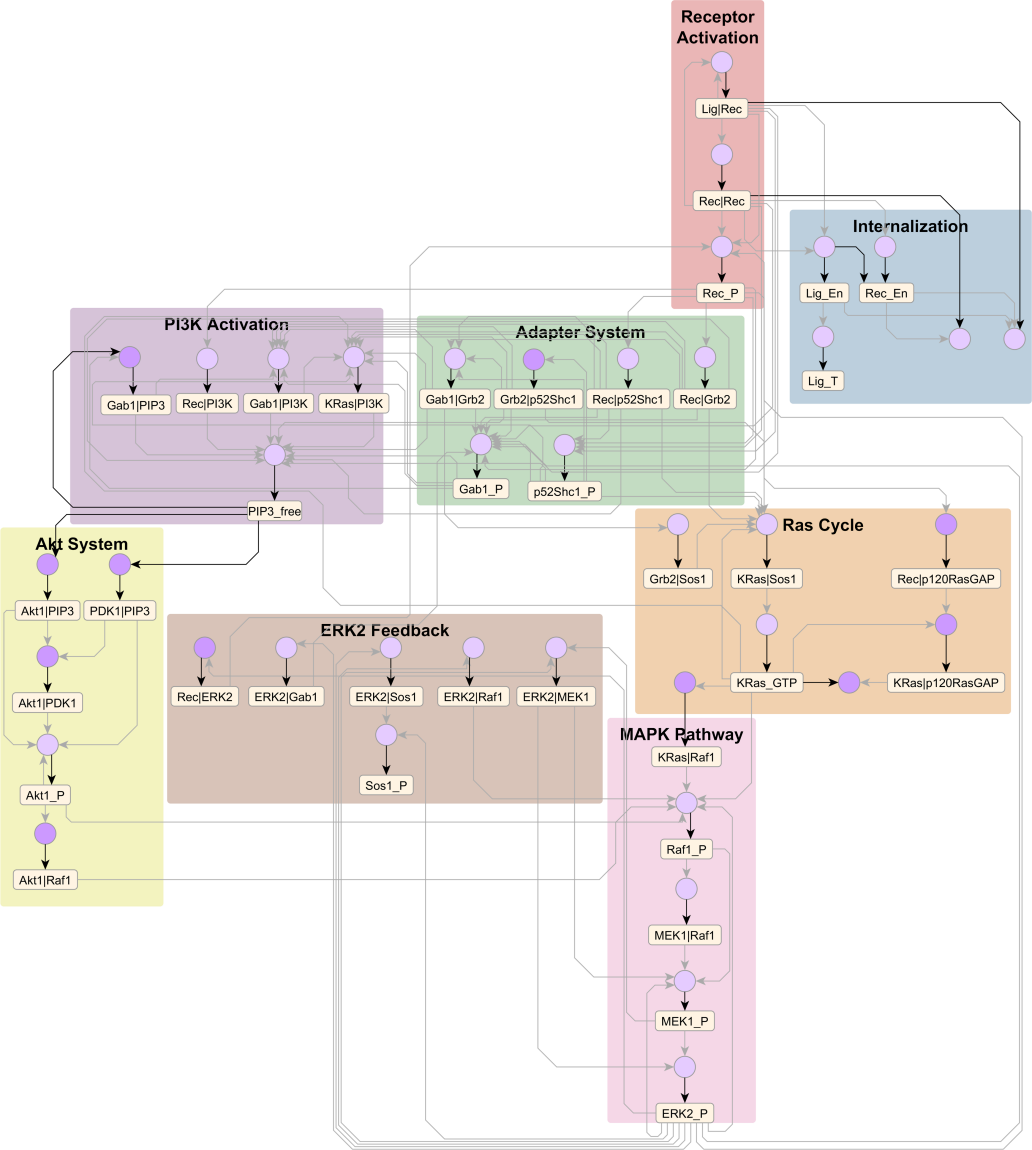
ErbB library of rules. Compressed AR graph (79 nodes, 144 edges) generated from the ErbB model of Creamer et al. [14]) with 625 rules. The uncompressed graph has 930 nodes and 5269 edges. The model elements can be roughly classified into eight subsystems shown above. The files needed to reproduce this diagram are provided in S1 Dataset.

To test the scaling of our approach to the growing set of large rule-based models [10,12,14,16], we applied the AR graph compression pipeline to two extensive models of receptor signaling: the FcεRI rule library constructed by Chylek et al. [10] (17 molecule types, 178 rules), and the ErbB signaling model constructed by Creamer et al. [14] (19 molecule types, 625 rules). The compressed graphs for these libraries are shown in Figs 7 and 8 respectively. Unlike the manually constructed Extended Contact Maps (ECMs) [23] that were published with these models, the graphs we show are pathway diagrams that were generated directly from the model specification. In S2 Appendix, we provide a tutorial on generating similar diagrams using the yeast pheromone signaling model of Suderman and Deeds [40] (26 molecule types, 272 rules) as an example.

The modeler can customize pipeline inputs to capture specific biochemical features in the model as well as strike a balance between compression and resolution on the output graph. For example, the default heuristic assumes that co-occurring phosphorylation sites can be grouped together, but for the FcεRI model, we wanted to distinguish between co-occurring phosphorylation sites with opposing functions, specifically those on Src family kinases Lyn and Fyn (SFKs). So, during atom grouping, we grouped functionally similar sites across molecules, e.g., the group SFK_Act_p contains activation-related phosphorylation sites on both Lyn and Fyn. As a result, the output graph (Fig 7) resolves the regulatory interactions of a generic SFK rather than Lyn and Fyn individually. Similarly, for the much larger ErBb model, creating functional groups such as ligands, receptors, and receptor dimers caused a dramatic reduction in complexity, with the output graph (Fig 8) showing signaling interactions of a generic ErbB receptor. Alternatively, grouping Lyn sites separately from Fyn or EGFR and ErbB2 receptors separately from ErbB3 and ErbB4 will produce graphs larger than those shown in Figs 7 & 8, with regulatory interactions resolved in more detail.

**Fig 9:**
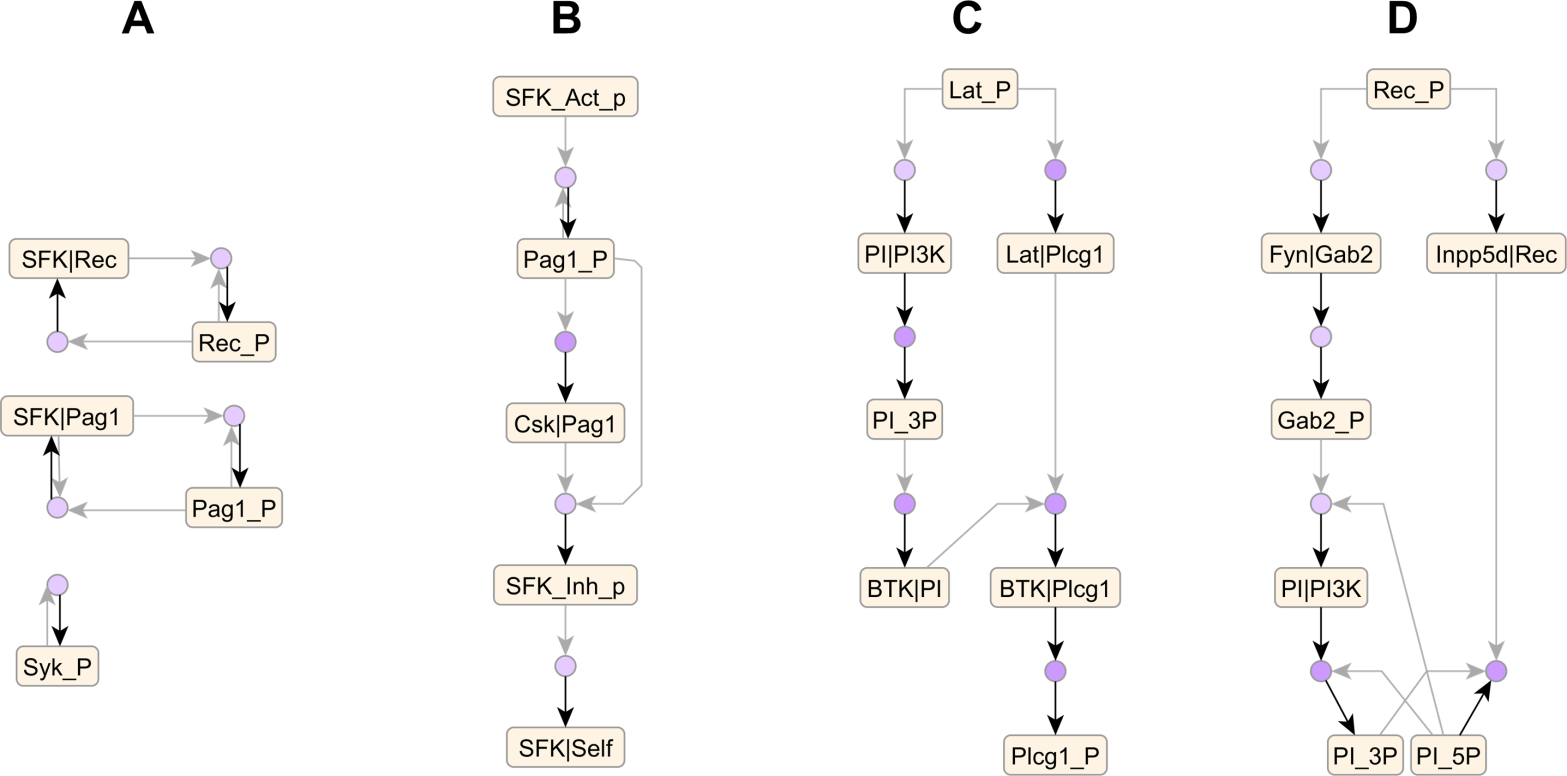
Signaling motifs in the FcεRI library recovered from the compressed AR graph. (A) Positive feedback loops enhance binding of Src family kinases Lyn and Fyn (denoted SFK) to receptor and Pag1 scaffold as well as Syk auto-phosphorylation. (B) Pag1 phosphorylated by SFKs recruits Csk, which negatively regulates SFKs by phosphorylation. (C) A coherent feed-forward loop activates Plcg1 from phosphorylated Lat. (D) An incoherent feed-forward loop involving enzymes PI3K and Inpp5d (a.k.a. SHP2) regulates levels of phosphoinositide PIP3, which is phosphorylated at both 3’ and 5’ hydroxyl positions (denoted PI_3P and PI_5P respectively).

The compressed AR graph offers a convenient venue for analysis and exploration of a rule-based model. For example, on the FcεRI and ErbB graphs, we were able to identify well-known pathways such as MAPK (transparent overlays in Figs 7 & 8) using a combination of node clustering and visual inspection. Also, on the FcεRI graph, we were able to trace network motifs encoded in the model (Fig 9) by Chylek *et al*. [10]. Without the compressed AR graph, the same analyses would have required examining hundreds of complex rules in various combinations, which would have required significant effort. Thus, the compressed AR graph offers a useful proxy for the rule-based model that is more amenable to analysis.

### Comparing complexity of visualization tools

**Fig 10:**
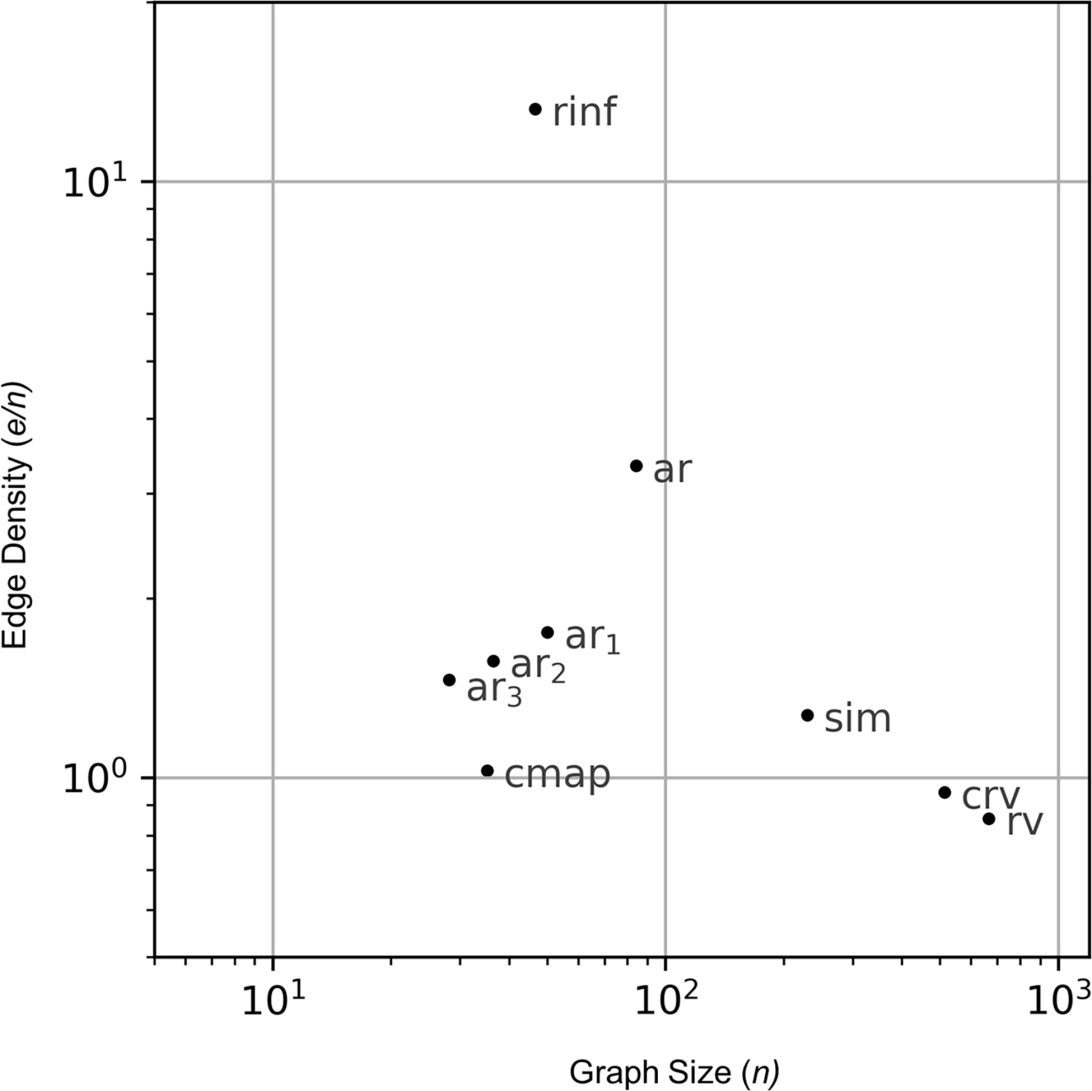
Comparison of readability metrics across visualization methods. Graph size and edge density averaged over 27 models (geometric mean) for 9 automated visualizations: contact map (cmap), conventional rule visualization (rv), compact rule visualization(crv), Simmune Network Viewer diagram (sim), rule influence diagram (rinf), full model atom-rule graph (ar), AR graph with background removed (ar_1_), AR graph compressed with strict edge signature (ar_2_) or permissive edge signature (ar_3_). The compression pipeline (ar_1-3_) is semi-automated, but here it was applied automatically with default settings. The full dataset is shown in S3 Fig.

To assess the readability of various visualization tools, we examined the joint distribution of graph size *n* and edge density *e/n* for each visualization when applied to 27 published rule-based models (see Methods), where *n* and *e* refer to number of nodes and edges respectively. In S3 Fig, we report these distributions for 9 visualization methods, and in Fig 10, we show their geometric means. The choice of metrics follows from Ghoniem *et al*. [43], who determined that user performance on visual graph analysis tasks decays with increasing graph size and edge density. Ghoniem *et al*. used much denser graphs than the ones in our test set, so we replaced their edge density metric √*(e/n*^*2*^*)* with *e/n*, which has a higher coefficient of variation for the graphs in our test set (2.54 vs 1.03), and therefore higher discriminatory power.

The results in Fig 10 confirm our qualitative observations on the readability of current visualizations and the improvements present in our new ones. Contact maps are generally compact with sparse edges as they only show structural composition and do not show individual mechanisms or signal flow. Rule visualizations, both conventional and compact, produce large graphs with sparse edges as they show the patterns encoded in each rule. However, compact rule visualizations are smaller than conventional ones as they make use of graph operation nodes. Diagrams showing interactions of rules are typically dense, such as rule influence diagrams and full AR graphs. However, full AR graphs have much lower edge density than rule influence diagrams as they use atoms to mediate interactions between rules. When the compression pipeline is applied, AR graphs’ size and edge density can be reduced to approach that of contact maps. This makes compressed AR graphs as readable as contact maps, while conveying substantially more information about the signaling architecture. The Simmune Network Viewer, which is intermediate between rule visualizations and full AR graphs, is discussed in detail below.

## Discussion

In this work we have developed new visualization approaches for rule-based models. The novel compact rule visualization conveys the mechanism underlying individual rules more effectively than conventional visualizations. The atom-rule (AR) graph conveys interactions between rules more efficiently than rule influence diagrams. A compression pipeline for the AR graph flexibly accounts for nuances of specific biological systems and reproducibly generates compact pathway diagrams even for models with hundreds of complex rules. These tools open the door for new forms of analysis for rule-based models such as network motif identification. In Supplementary Material, we show the theoretical foundation for these tools (S1 Appendix) as well as a tutorial for how to apply them to a large rule-based model (S2 Appendix).

### Philosophical perspective

Edward R. Tufte, a pioneer of modern data visualization and analytic design, argues that “universal cognitive tasks” underlie how humans perceive information and motivates that “cognitive tasks should be turned into design principles” [44]. In the biochemical literature, diagrams and text employ a number of such cognitive tasks, and our automated methods recapitulate some of these. For example, one often describes a biochemical process using an action verb such as “binds” or “phosphorylates”. Graph operation nodes in compact rule visualization (Fig 2D) play a similar role in conveying the action of a rule. Similarly, one uses “site” to denote a molecular part that behaves distinctly or is targeted by a specific process. Atoms used in the atom-rule graph (Fig 3A) have a similar interpretation as types of actionable sites. Literature descriptions and diagrams also selectively emphasize active states over ground states and signal-activated processes over processes that attenuate the signal or occur in the background, which allows the reader to filter redundant information. Removing low priority nodes on the AR graph follows a similar principle (Fig 4B). Text descriptions routinely categorize molecules and sites using principles such as homology and functional similarity [45–48], and use broad terms to summarize information about specific molecules and sites. Grouping atoms and rules using the described heuristics (Fig 4C–4D) and compressing the AR graph (Fig 4E) recapitulates this approach.

### Caveats

Whenever compression is applied to data, there exists a many-to-one relationship between the uncompressed and compressed representations. In the context of visualization, a rule-based model will generate the same conventional and compact rule visualizations and *vice versa*, but different models can generate the same contact map, rule influence diagram and AR graph. Therefore, one should use each tool at the resolution for which it is designed to be used. Compact rule visualization should be used to show the mechanism underlying each rule. The AR graph is less useful for this purpose, as it approximates each rule as a bipartite graph. Instead, it should be used to infer interactions between rules through formal or informal approaches. When applying the compression pipeline to the AR graph, one should verify that the choice of inputs is biologically reasonable. If this is the case, then the compressed AR graph is useful for both communicating the model to others as well as graph analysis.

### Related work

**Fig 11:**
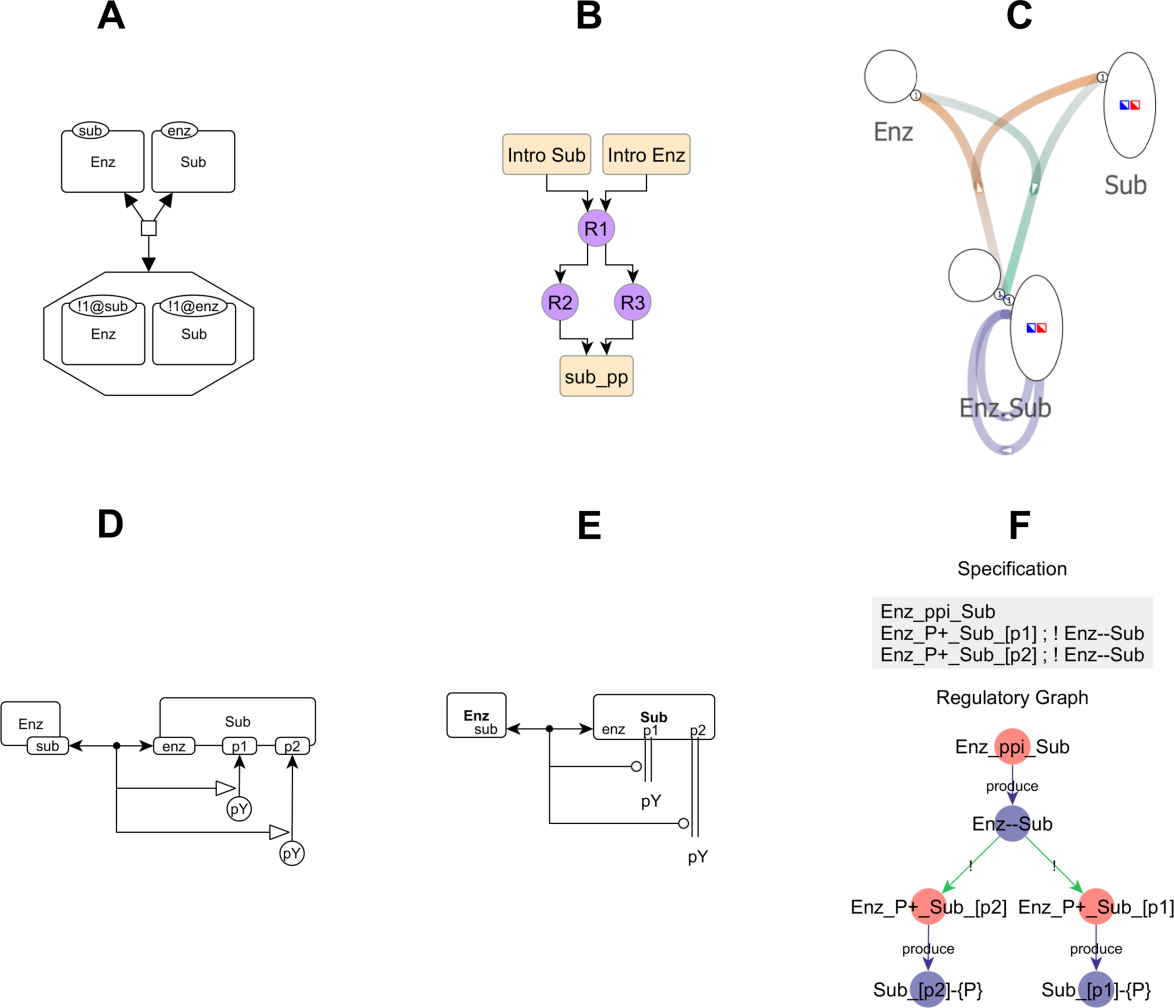
Other visualization approaches applied to the enzyme-substrate phosphorylation model of Fig 2. (A) The binding rule drawn using SBGN Process Description conventions, which require visual graph comparison. (B) Kappa story, showing the causal order of rules that produces sub_pp, which refers to doubly phosphorylated substrate. (C) Simmune Network Viewer diagram, which merges patterns across rules and hides certain causal dependencies (details in S4 Fig). Here, the Enz.Sub node merges all enzyme-substrate patterns shown in Fig 2. (D) SBGN Entity Relationship. (E) Molecular Interaction Map. Panels D-E require manual interpretation of the model, like the extended contact map. (F) *rxncon* regulatory graph visualization of the *rxncon* model format, which can only depict a limited subset of reaction rules (details in S5 Fig).

In addition to the approaches discussed in Introduction (Fig 1A–1D) and Methods (Fig 2C), we show examples of other currently available tools (Fig 11) and how they compare with compact rule visualizations and atom-rule graphs.

The SBGN Process Description (Fig 11A) [24] is a visualization standard for reacting entities. It has the same limitation as conventional rule visualization, namely the need for visual graph comparison.

The Kappa story (Fig 11B) [22] shows the causal order in which rules can be applied to generate specific outputs, and these are derived by analysis of model simulation trajectories. It is complementary to the statically derived AR graph for showing interactions between rules, but it does not show the structures that mediate these interactions nor does it provide a mechanism for grouping rules. Integrating Kappa stories with AR graphs is an interesting area for future work.

The Simmune Network Viewer (Fig 11C) [26] compresses the representation of rules differently from the AR graph: it merges patterns that have the same molecules and bonds, but differ in internal states. Like the AR graph, it shows both structures and rules, and it produces diagrams with much lower density (‘sim’ in Fig 10), but it obscures causal dependencies on internal states (S4 Fig).

The SBGN Entity Relationship diagram (Fig 11D) [24] and the Molecular Interaction Map (Fig 11E) [25], like the Extended Contact Map [23], are diagrams of model architecture that rely on manual analysis.

The *rxncon* regulatory graph (Fig 11F) visualizes the *rxncon* model format [27], which uses atoms (called elemental states in *rxncon*) to specify contextual influences on processes. This approach, which is also followed in Process Interaction Model[49], is less expressive than the graph transformation approach used in BioNetGen, Kappa and Simmune (S5 Fig). The AR graph we have developed generalizes the regulatory graph visualization so it can be derived from arbitrary types of rules found in BioNetGen, Kappa and Simmune models.

### Future work

The AR graph offers many advantages over existing methods, but there are a number of ways in which it could be improved or generalized. There are alternate ways to show the content of the AR graph, for example, as a two-dimensional matrix [43]. The compression algorithms can be extended to identify more complex relationships, for example, treating the consumption of an active state as an ‘inhibits’ relationship, grouping enzyme-binding and catalytic processes together as a Michaelis-Menten mechanism, etc. In the immediate future, we plan to add support for other features present in the BioNetGen model specification, such as compartmental states, transport rules and dependencies encoded in rate laws [50,51].

Additionally, the AR graph opens up rule-based models to a wide variety of analysis and visualization tools, as it transforms a complex rule-based model into a simple bipartite graph. For example, simulation fluxes can be conveniently visualized on a bipartite graph by mapping numeric values to node size or edge thickness [52]. Also, as *rxncon* developers have shown, one can perform stochastic Boolean simulations on a bipartite graph [53]. Model reduction approaches developed for rule-based models have previously used information on interactions between structures and rules [54] that can now be obtained directly from the AR graph. The AR graph also serves as a rich source of information that could be mined using formal approaches. Potential areas where new methods can be developed include identifying model subsystems (as in Figs 7 and 8) by graph partitioning [55], identifying network motifs (as in Fig 9) by cycle detection [56], dynamically grouping atoms and rules using graph structure discovery [57,58], etc. Thus, adoption of the AR graph could pave the way for novel applications of graph analysis, data mining and machine learning to rule-based models.

### Outlook

A natural future direction for signaling models is to explore the effects of complex input stimuli and crosstalk between pathways [59,60] on a comprehensive scale. This would require integrating rules from multiple sources, such as databases constructed in tandem by different groups (e.g. [10,12,14,34]). The recently published whole cell model of *Mycoplasma genitalium* [17] makes effective use of databases to organize and visualize kinetic information [61–63] and provides proof-of-concept of a database-oriented approach. Currently, models of signaling from various receptors have as many as hundreds of rules [10,12,14] and this number is expected to increase by an order of magnitude to cover more molecule types, receptors and signal pathways. We expect that AR graphs will play a role in the construction, navigation and visualization of the rule-based databases of the future, similar to approaches deployed on other biological data (VisANT [64], ChiBE [65]). The AR graph will also be useful for frameworks that implement rule-based data structures (SBML-Multi [33], BioPax Level 3 [66]) or integrate rules with higher-order model composition (Virtual Cell [18,19], PySB [34]). Thus, in addition to the immediate benefit of visualizing and understanding large models, the AR graph is expected to be useful in developing the comprehensive cell models of the future.

## Supporting information

S1 Fig Graph operation nodes. Supported graph operation nodes for compact rule visualization. AddBond and DeleteBond are placed adjacent to the pair of components on which a bond is added or removed respectively. AddMol and DeleteMol are placed adjacent to the molecule that is added or removed respectively. AddBond/AddMol nodes have edges pointed outward from the graph operation node to indicate that a new structure is created, whereas DeleteBond/DeleteMol nodes have edges pointed inward to indicate that an existing structure is destroyed. ChangeState node is placed adjacent to the internal state that is modified. It has one incoming edge from the initial state and one outgoing edge to the destination state. The labels of the graph operation nodes are hidden in the main text figures, but are evident from the edge directions.
(TIF)

S2 Fig Groups on the atom-rule graph. (A) On the full AR graph of Faeder et al. [9], the default heuristic groups phosphorylation sites on the same molecules (e.g., Rec_pY) and binding interactions between the same pairs of molecules (e.g., Lyn|Rec). Then, an algorithm groups rules that share the same edge signature, i.e., if they have the same edges to the same adjacent atom groups. (B) A strict edge signature accounts for all three edge types and resolves rule variants that have the same reactant/product edges but different context edges (e.g., R12 and R13), i.e., it does not group them together. (C) A permissive edge signature ignores context edges, which results in broadly defined groups (e.g., rules R10-R13) that do not resolve contextual rule variants. The labels of the rule nodes and rule group nodes are hidden in the main text figures.
(TIF)

S3 Fig Distribution of readability metrics for various visualization methods. Graph size and edge density of 27 rule-based models (blue) and their geometric mean (red) for 9 types of visualizations: (A) contact map, (B) conventional rule visualization, (C) compact rule visualization, (D) Simmune Network Viewer, (E) rule influence diagram, (F) full model atom-rule graph, (G) model AR graph with low-priority nodes removed, then (H) compressed using a strict edge signature, or (I) a permissive edge signature. The geometric means for each visualization type are also plotted in Fig 10.
(TIF)

S4 Fig Comparison of AR graph and simmune network viewer. **(A)** A model in which three sites on a protein are activated in sequence. **(B)** The sequence is evident on the AR graph. **(C)** The sequence cannot be seen on the Simmune Network Viewer diagram because the three patterns used have the same molecule stoichiometry {A = 1} and are represented by the same node, which obscures information mediated through state changes.
(TIF)

S5 Fig Comparison of AR graph and *rxncon* regulatory graphs. **(A)** In BioNetGen, complex reaction mechanisms are specified as reaction rules and the AR graph is inferred by analyzing the specified rules. The reaction rule shown models *trans*-phosphorylation of receptor R in the ligand-crosslinked dimer configuration by recruited kinase K, a frequently encountered mechanism in biochemical signaling. **(B)** In *rxncon*, regulation is specified using the *rxncon* syntax and directly visualized as the regulatory graph. Reaction mechanisms are reconstructed from the specified regulatory interactions and are limited to a small set of mechanisms, e.g., the current version of *rxncon* does not natively support *trans*-phosphorylation reactions.
(TIF)

S1 Appendix Detailed methods, algorithms and rendering conventions. (DOCX)

S2 Appendix Tutorial. (DOCX)

S1 Table List of models. (DOCX)

S1 Dataset Supplementary material for tutorial. (ZIP)

S2 Dataset Supplementary material for readability analysis. (ZIP)
