# Supplementary figures and images for: Automated visualization of rule-based models
Source: PLoS Comput Biol. 2017 Nov 13;13(11):e1005857. doi: 10.1371/journal.pcbi.1005857 (PMC5703574; doi:10.1371/journal.pcbi.1005857)

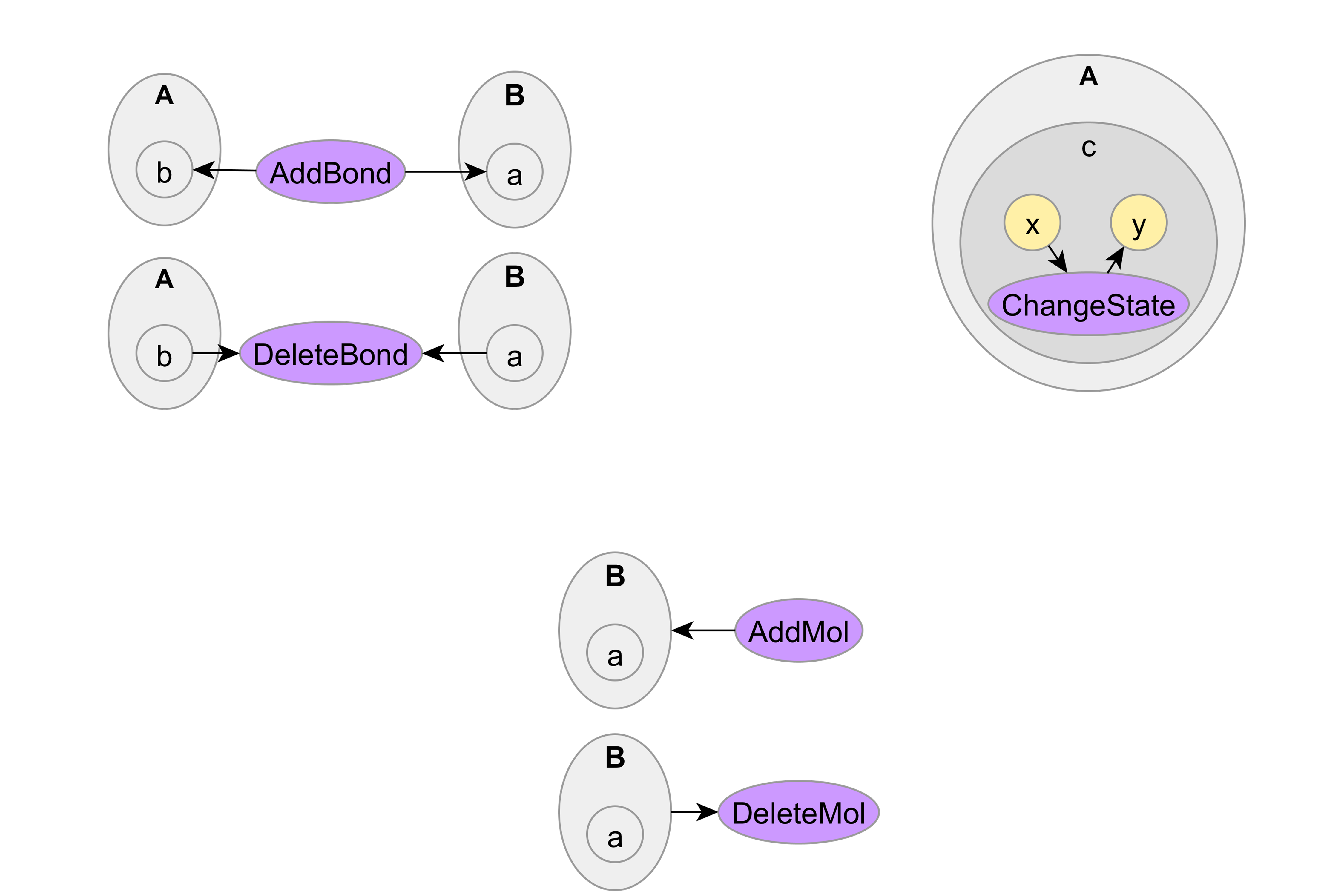

Supplement: S1 Fig — Supported graph operation nodes for compact rule visualization. AddBond and DeleteBond are placed adjacent to the pair of components on which a bond is added or removed respectively. AddMol and DeleteMol are placed adjacent to the molecule that is added or removed respectively. AddBond/AddMol nodes have edges pointed outward from the graph operation node to indicate that a new structure is created, whereas DeleteBond/DeleteMol nodes have edges pointed inward to indicate that an existing structure is destroyed. ChangeState node is placed adjacent to the internal state that is modified. It has one incoming edge from the initial state and one outgoing edge to the destination state. The labels of the graph operation nodes are hidden in the main text figures, but are evident from the edge directions. (TIF) [file pcbi.1005857.s001.tif]

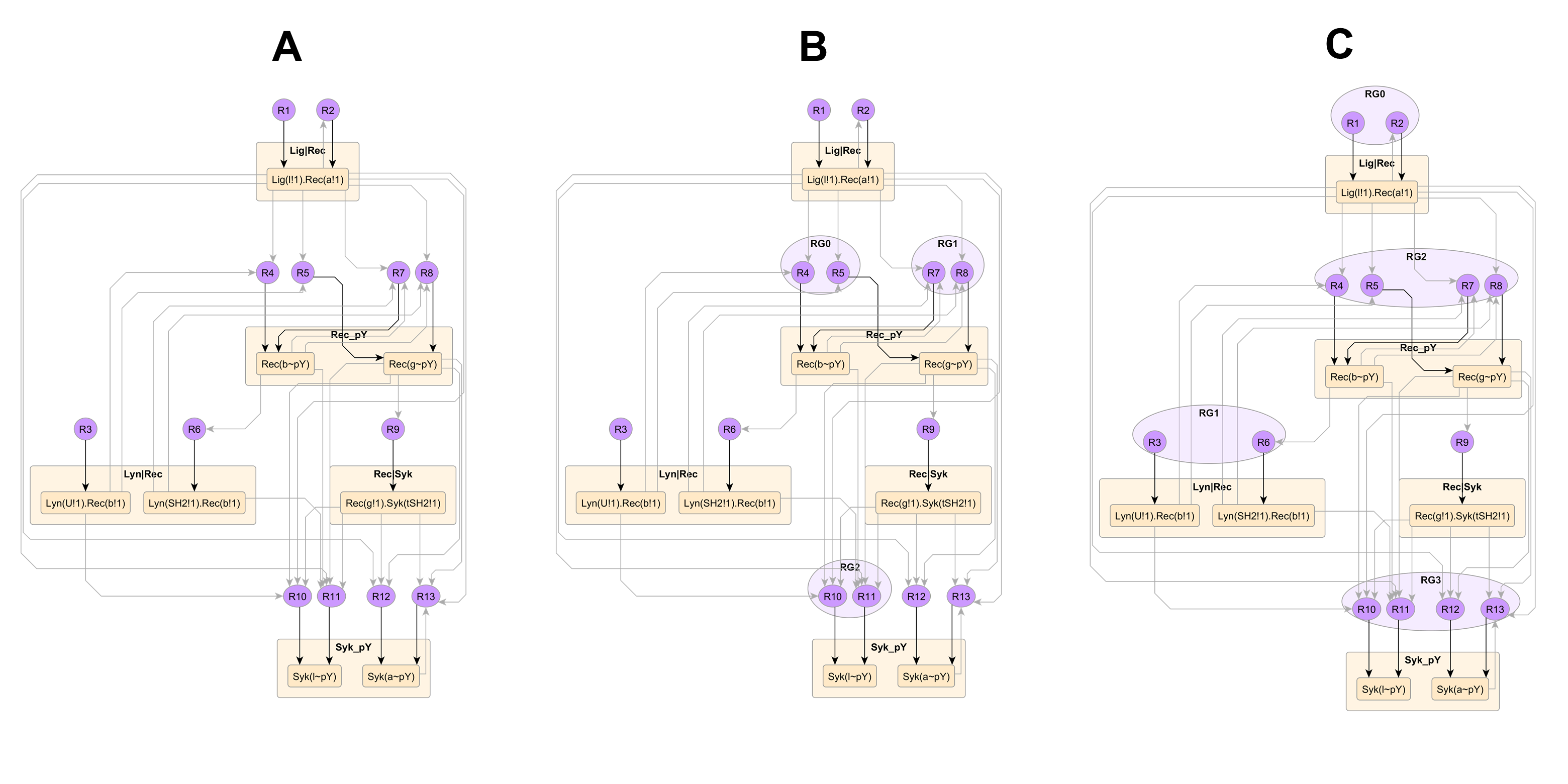

Supplement: S2 Fig — (A) On the full AR graph of Faeder et al. [9], the default heuristic groups phosphorylation sites on the same molecules (e.g., Rec_pY) and binding interactions between the same pairs of molecules (e.g., Lyn|Rec). Then, an algorithm groups rules that share the same edge signature, i.e., if they have the same edges to the same adjacent atom groups. (B) A strict edge signature accounts for all three edge types and resolves rule variants that have the same reactant/product edges but different context edges (e.g., R12 and R13), i.e., it does not group them together. (C) A permissive edge signature ignores context edges, which results in broadly defined groups (e.g., rules R10-R13) that do not resolve contextual rule variants. The labels of the rule nodes and rule group nodes are hidden in the main text figures. (TIF) [file pcbi.1005857.s002.tif]

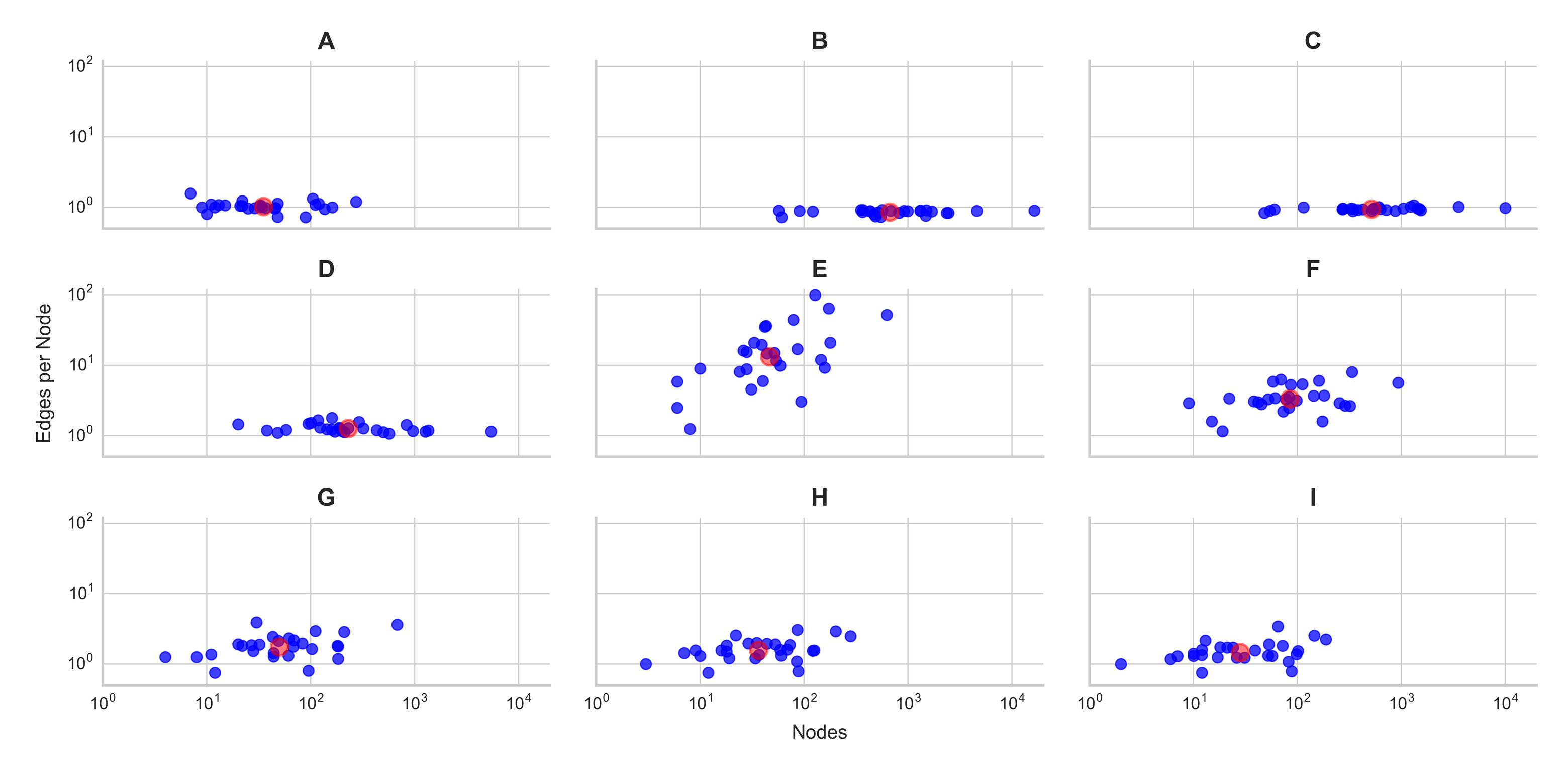

Supplement: S3 Fig — Graph size and edge density of 27 rule-based models (blue) and their geometric mean (red) for 9 types of visualizations: (A) contact map, (B) conventional rule visualization, (C) compact rule visualization, (D) Simmune Network Viewer, (E) rule influence diagram, (F) full model atom-rule graph, (G) model AR graph with low-priority nodes removed, then (H) compressed using a strict edge signature, or (I) a permissive edge signature. The geometric means for each visualization type are also plotted in Fig 10. (TIF) [file pcbi.1005857.s003.tif]

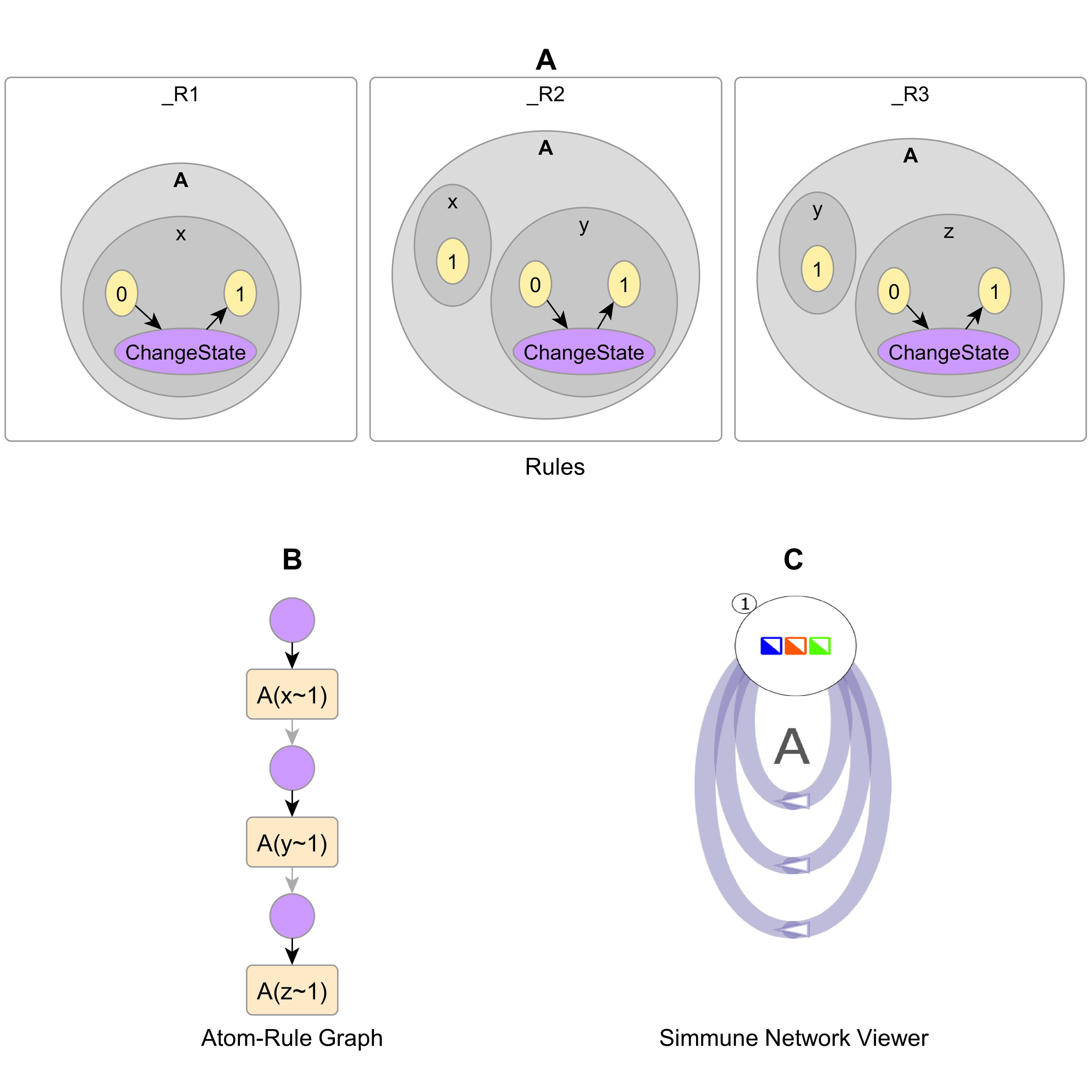

Supplement: S4 Fig — (A) A model in which three sites on a protein are activated in sequence. (B) The sequence is evident on the AR graph. (C) The sequence cannot be seen on the Simmune Network Viewer diagram because the three patterns used have the same molecule stoichiometry {A = 1} and are represented by the same node, which obscures information mediated through state changes. (TIF) [file pcbi.1005857.s004.tif]

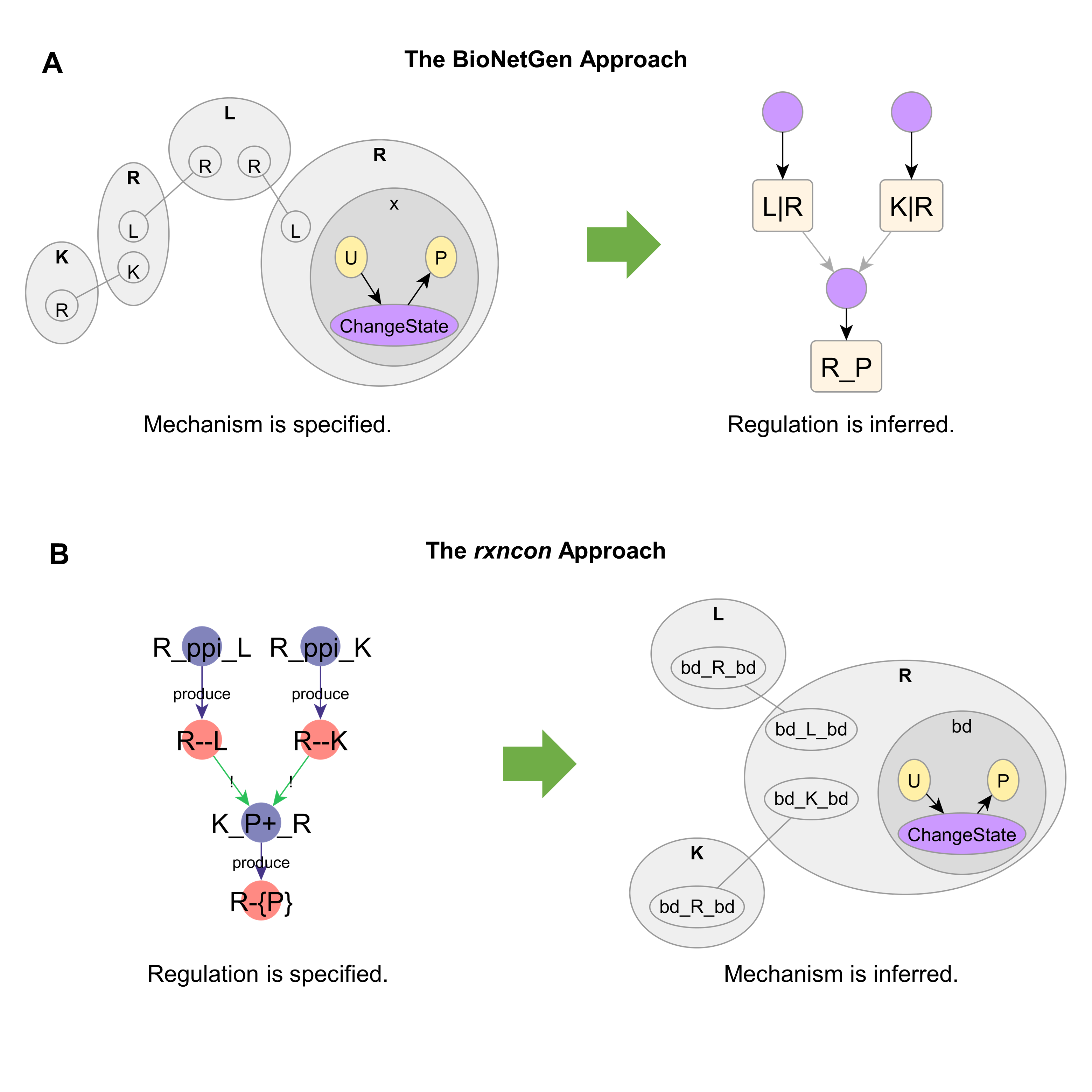

Supplement: S5 Fig — (A) In BioNetGen, complex reaction mechanisms are specified as reaction rules and the AR graph is inferred by analyzing the specified rules. The reaction rule shown models trans-phosphorylation of receptor R in the ligand-crosslinked dimer configuration by recruited kinase K, a frequently encountered mechanism in biochemical signaling. (B) In rxncon, regulation is specified using the rxncon syntax and directly visualized as the regulatory graph. Reaction mechanisms are reconstructed from the specified regulatory interactions and are limited to a small set of mechanisms, e.g., the current version of rxncon does not natively support trans-phosphorylation reactions. (TIF) [file pcbi.1005857.s005.tif]

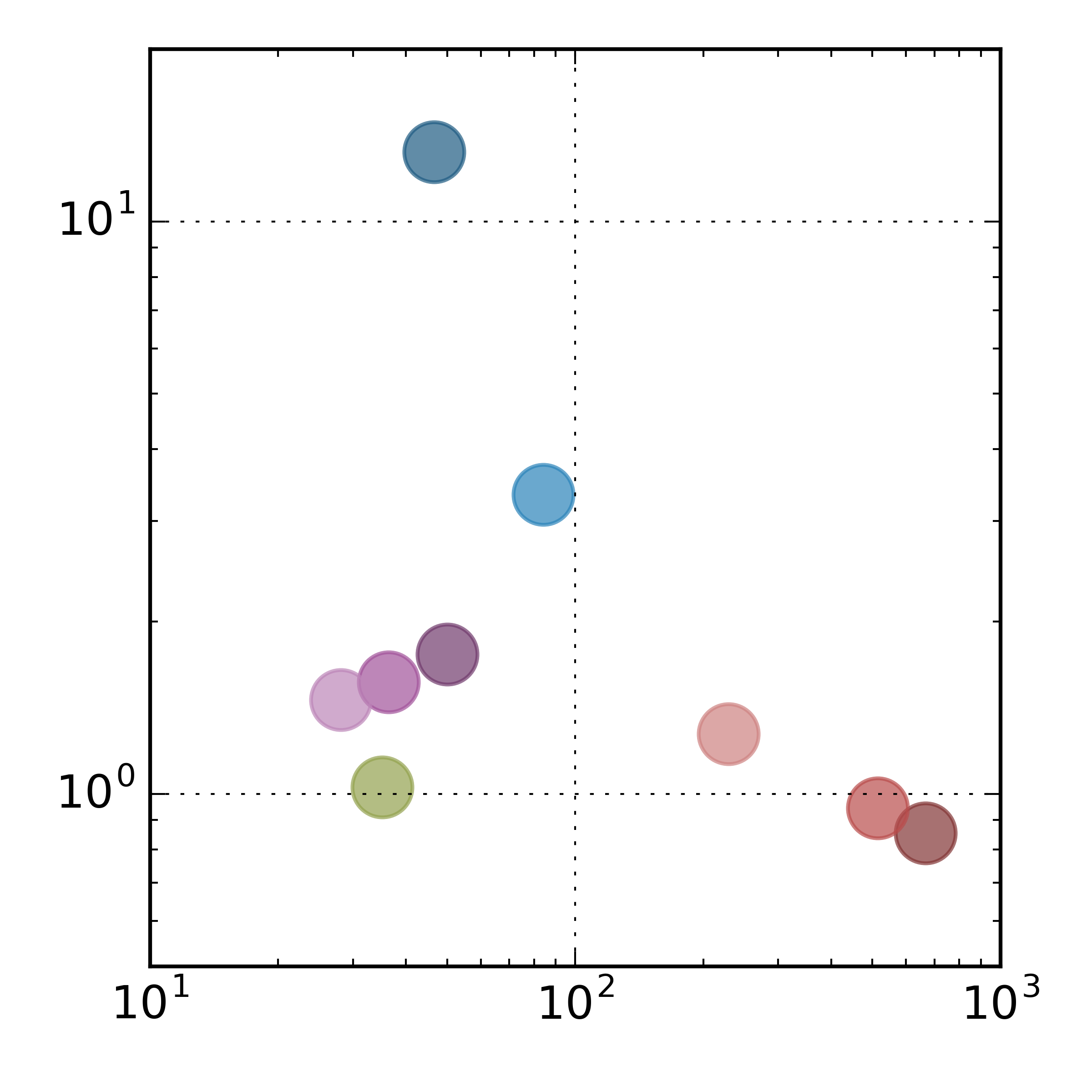

Supplement: S2 Dataset — (ZIP) [file pcbi.1005857.s010.zip › model_analysis/data/gmeans.png]

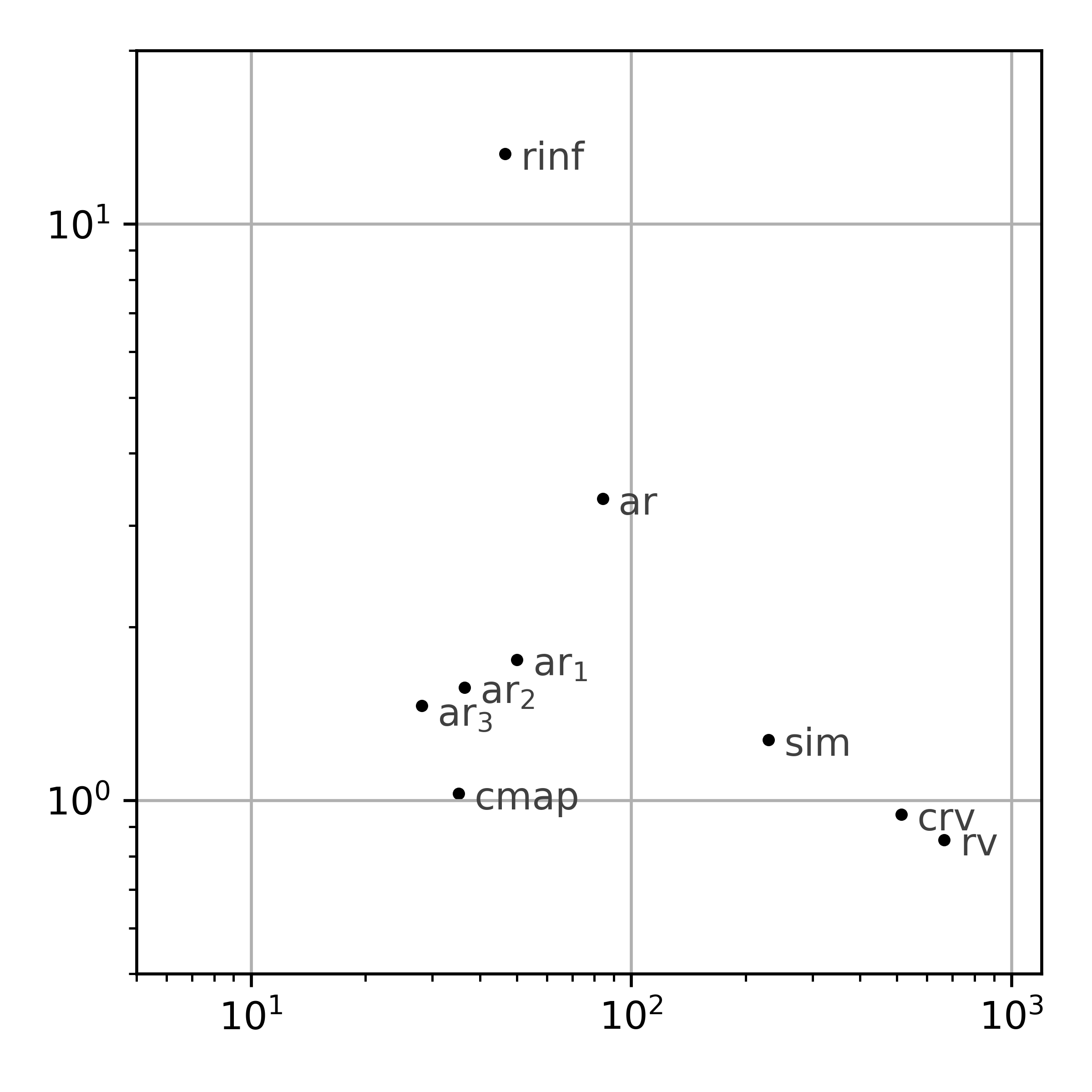

Supplement: S2 Dataset — (ZIP) [file pcbi.1005857.s010.zip › model_analysis/data/gmeans2.png]

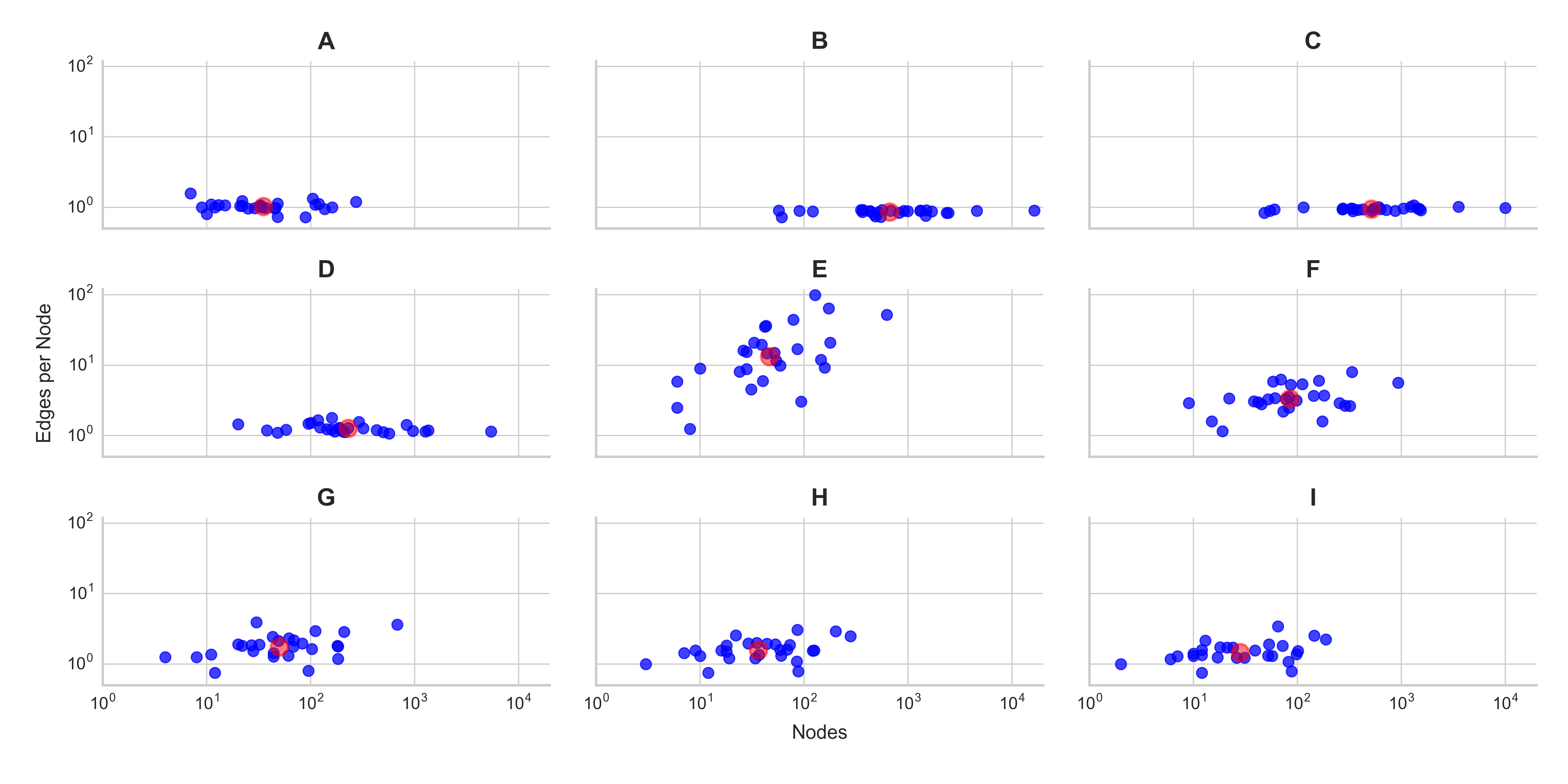

Supplement: S2 Dataset — (ZIP) [file pcbi.1005857.s010.zip › model_analysis/data/modelgraphs.png]

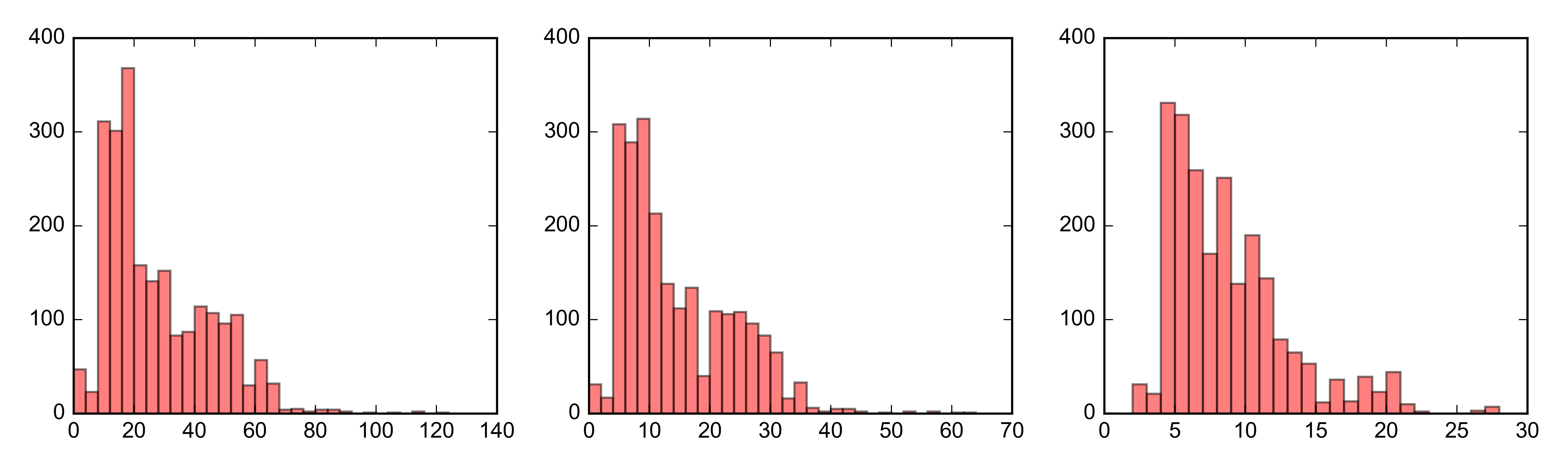

Supplement: S2 Dataset — (ZIP) [file pcbi.1005857.s010.zip › model_analysis/data/rulegraphs.png]

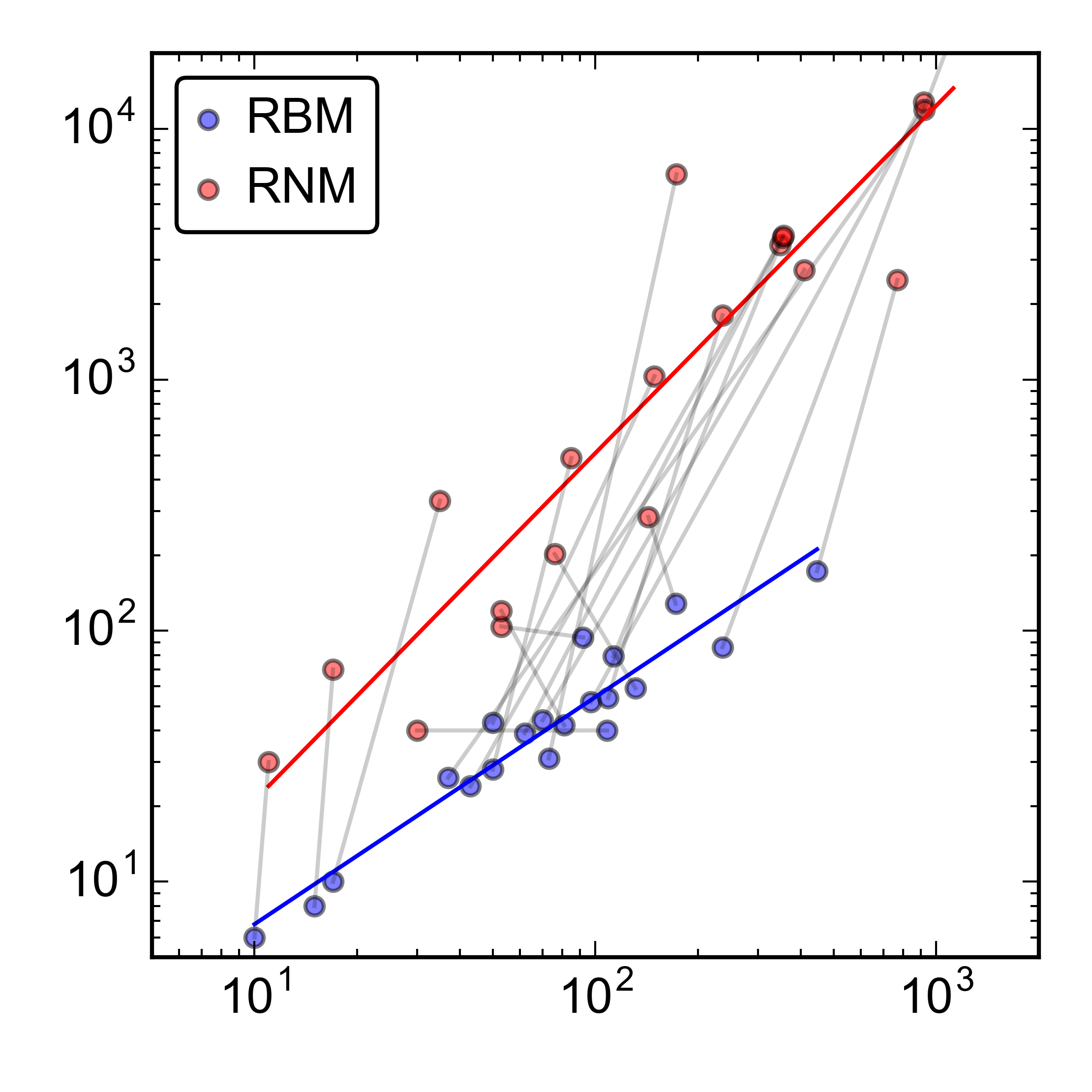

Supplement: S2 Dataset — (ZIP) [file pcbi.1005857.s010.zip › model_analysis/data/rxnnet.png]
