# Supplementary material for: Automated visualization of rule-based models
Source: PLoS Comput Biol. 2017 Nov 13;13(11):e1005857. doi: 10.1371/journal.pcbi.1005857 (PMC5703574; doi:10.1371/journal.pcbi.1005857)
Supplement: S1 Appendix — (DOCX) [file pcbi.1005857.s006.docx]

S1 Appendix: Extended Methods

# Index

S1.1: Extended methods
 Approach
 Intermediary Graph Types
 Visualizing Structure
 Visualizing Mechanism
 Visualizing Regulation
 Visualizing Function
 Algorithm Time Complexity
 Heuristics for Background Removal
 Heuristics for Atom Grouping
 Figures
 F1: Summary of Methods
 F2: Intermediary Graphs and Conventional Visualizations
 F3: Compact Rule Visualization
 F4: Atom-Rule Graph
 F5: Complexity Reduction Approaches
 F6: Rule size distributions
S1.2: Algorithms
 A1: Pattern Structure Graph
 A2: Correspondence Map
 A3: Rule Structure Graph
 A4: Atom-Rule (AR) Graph
 A5: Model AR Graph
 A6: Background Removal
 A7: Edge Signature of a Rule
 A8: Grouping Atoms and Rules
 A9: Collapsing Groups
S1.3: Rendering Conventions
 C1: Site Graph
 C2: Compact Rule Visualization
 C3: AR Graph

S1.1: Extended Methods

**Approach:** The goal of this work is to formally derive useful visualizations of rule-based models. The input for these methods include BioNetGen patterns and rules, whose formal definitions have been provided in previous literature (see Supplement in [1]). These are converted to intermediary graphs, which are graph data structures defined in this work. Diagrams are generated by applying rendering conventions to the nodes and edges of the intermediary graphs. Fig F1 provides a broad summary. In this section, we briefly discuss the data structures, algorithms and rendering conventions. For detailed specifications on algorithms and rendering conventions, refer to Sections S7.2 and S7.3 respectively.


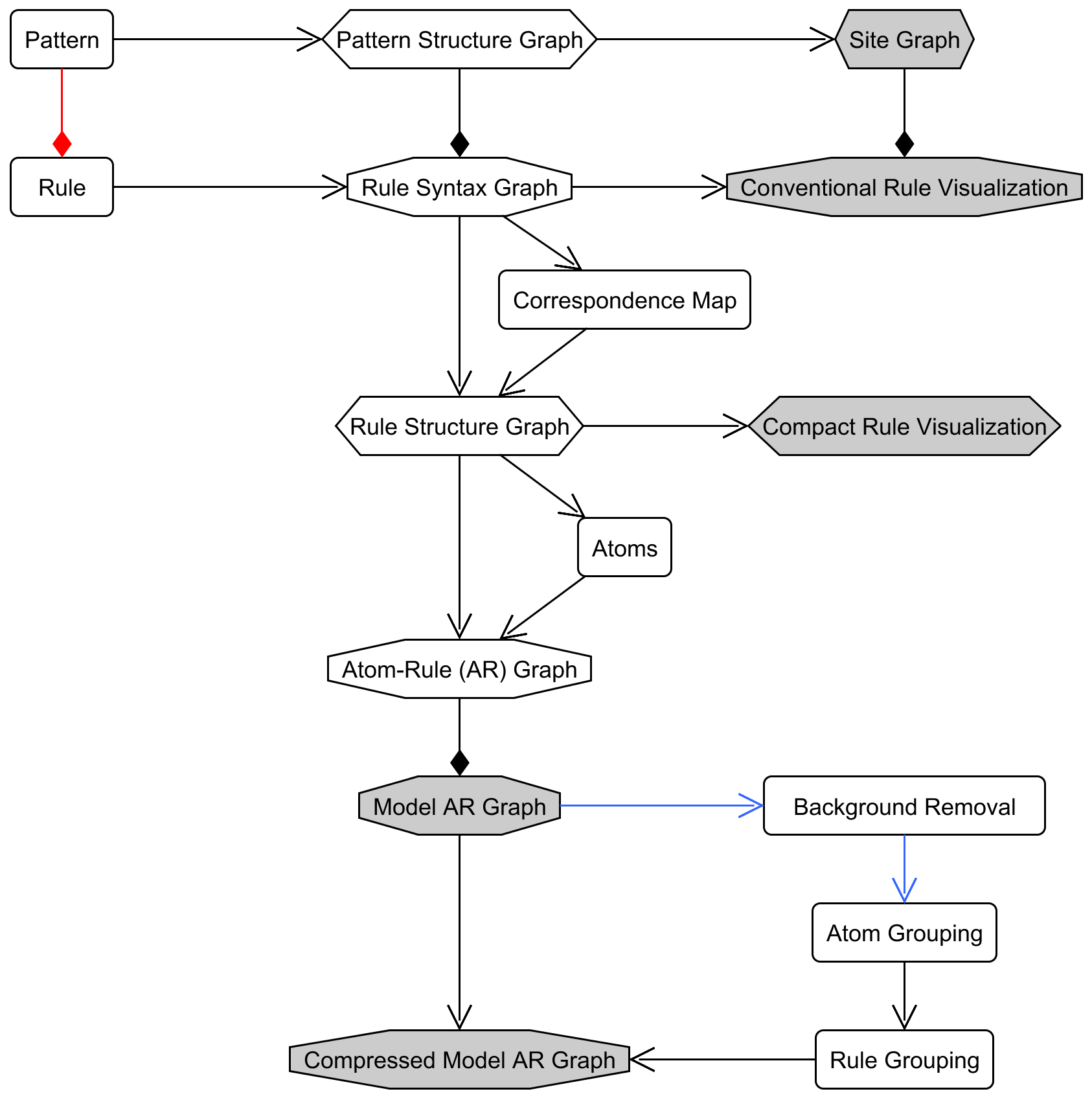


**Fig F1 Summary of methods.** The graphs in this work are of two main types, structure graph (hexagon) or network graph (octagon). Objects are either transformed or generated from other objects (open arrowhead), or composed from multiple instances of other objects (diamond arrowhead). Each process is either user-specified (red), automated (black) or semi-automated (blue). In the model specification, patterns compose rules. For visualization, they are transformed to pattern structure graphs and rule syntax graphs respectively, which are visualized along conventional lines. In this work, we use the correspondence map synthesized from the rule syntax graph to create the rule structure graph, which can be visualized compactly. Also, the rule structure graph is examined to identify instances of atoms, which are then used to build the atom-rule (AR) graph. AR graphs of individual rules are aggregated into the model AR graph. Complexity reduction techniques (background removal, atom grouping, rule grouping and group collapsing) are sequentially applied to generate the compressed model AR graph.

**Intermediary Graph Types:** Intermediary graphs are of two broad types: structure graphs and network graphs (Fig F2A). **Structure graph** denotes a node-labeled undirected graph, with node attributes including but not limited to { NodeIndex, NodeLabel, NodeType }. Multiple nodes with the same NodeLabel and NodeType are allowed, but not with the same NodeIndex. Structure graphs are used to represent objects with predominantly structural information. **Network graph** denotes an edge-labeled node-labeled bipartite graph. Node attributes are { NodeLabel, NodeType } with NodeLabel being used to uniquely identify nodes. Network graphs are allowed to have only two node types with one node type referring to a structure and the other to a process. Edges are bipartite, i.e. they can only be from a structure node to a process node and not between the same node types. Edges on the network graph have one or more binary attributes that are typically mapped to visual attributes such as color and direction. Network graphs are used to represent the interaction of processes with structures. Structure graphs and network graphs have precedent in hierarchical graphs [2] and *rxncon* regulatory graphs [3] respectively.


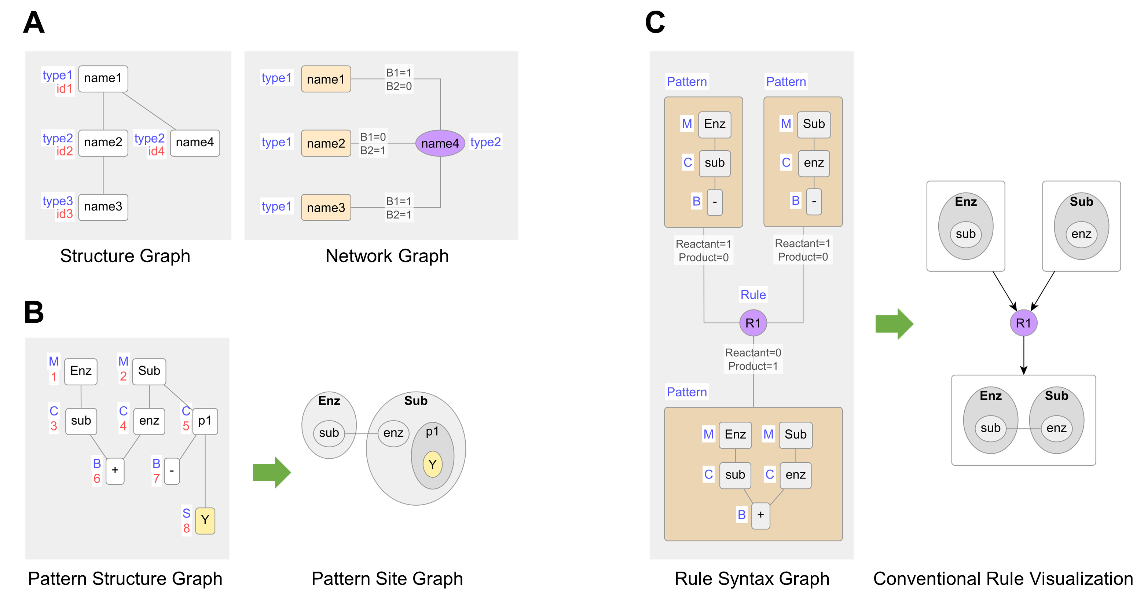


**Fig F2. Intermediary graphs and conventional visualizations. (A)** Structure graphs ({NodeIndex, NodeLabel, NodeType} as node attributes) and network graphs ({NodeLabel, NodeType} as node attributes) are the two types of graph data structures used in this work. Edges on the network graph are bipartite and have one or more edge attributes that can take binary values. **(B)** Patterns are represented by the pattern structure graph, which has molecule, component, binding state and internal state as values for NodeType (denoted M, C, B, S respectively). To visualize a pattern as a site graph, hierarchically nest molecules (nodes 1,2), components (3,4) and internal states (8), show bonds (6) as edges between components, and ignore unbound states (7) **(C)** The rule as specified in the model is represented by the rule syntax graph, a network graph in which one node type embeds pattern structure graphs and the other is labeled with a rule name. To produce a conventional rule visualization, render embedded patterns as site graphs and use edge direction to show reactant and product relationships.

**Visualizing Structure:** The **pattern** is the BioNetGen object that is used to specify molecular structures. It is built from molecules, components of molecules, internal states of components and bonds between components [1], [4], [5]. Here, we represent the pattern as the **pattern structure graph,** in which each molecule, component, internal state and bond state (bond or unbound state) is represented as a node (Fig F2B, Algorithm A1) ([1], [2], [6] have similar graph formalisms) . To visualize the pattern as a **site graph** (Fig F2B), nest molecules, components and internal states hierarchically and show bonds as edges between components (Rendering Convention C1). The chemical species and the contact map are both special cases of the pattern structure graph and have the same rendering conventions.

**Visualizing Mechanism**: The **reaction rule** is the BioNetGen object that is used to specify kinetic processes. It contains patterns specified as reactants and products [1], [4], [5]. The **rule syntax graph** is a network graph in which one type of node embeds pattern structure graphs, the other type of node is labeled with a rule name, and edges between the nodes are typed as reactant or product (Fig F2C). This encodes the reaction rule as it is specified in the model. To achieve a **conventional rule visualization** (Fig F2C), render the embedded patterns as site graphs and use edge direction to show whether the relationship is reactant or product (pattern to rule = reactant, rule to pattern = product).

To achieve a **compact rule visualization** (Fig F3A), we first build a correspondence map between left and right sides of the rule (Algorithm A2). Then, we merge the shared structures between reactant and product sides of the rule to produce a single graph called the **rule structure graph** (Algorithm A3). The resultant nodes inherit the property of whether they originate from reactant side or product side or both, which is represented as the attribute NodeSide. This explicitly differentiates modified structures from unmodified ones. Now, render unmodified nodes on the rule structure graph using site graph conventions and use graph operation nodes to emphasize the modified structures (Rendering convention C2). Five types of graph operations are permitted in BioNetGen rules (Fig F3B): adding and removing bonds (AddBond/DeleteBond), creating and destroying whole molecules (AddMol, DeleteMol) and changing the internal state label of a component (ChangeState). A rule may have more than one graph operation occurring simultaneously. Edges on the graph operation node also indicate whether the operation consumes or produces structures.


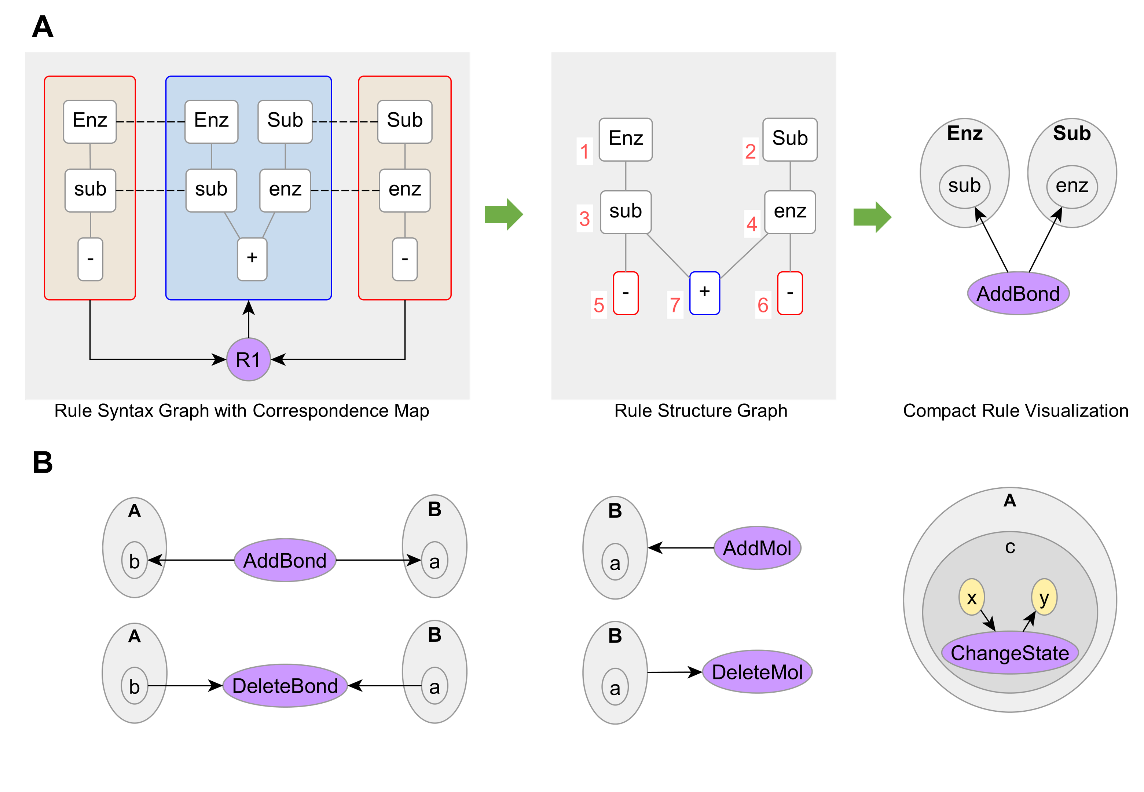


**Fig F3. Compact rule visualization. (A)** Given a rule syntax graph, BioNetGen maps out the shared structures between reactants and products by building a correspondence map (dashed lines). We use the correspondence map to merge reactants and products into a single rule structure graph. Nodes on this graph are also labeled with a side of origin: reactant only (red node border), product only (blue) or both (gray). To visualize rules compactly, render the rule structure graph as a site graph (nodes 1-6) and use graph operation nodes (AddBond) to emphasize the modified structures (node 7). **(B)** BioNetGen has five basic graph operations: adding and removing bonds, creating and deleting molecules and changing state labels of molecular sites. A rule may have one or more instances of these graph operations. In the main text, we hide labels of operation nodes.

**Visualizing Regulation:** We introduce the notion of **atom**, a special type of pattern that specifies only a single distinct type of modifiable structure. Atoms include free binding sites and bonds (modifiable by AddBond/DeleteBond), types of molecules (modifiable by AddMol/DeleteMol) and internal states (modifiable by ChangeState). Atoms are determined by examining specific source nodes on rule structure graphs (Fig F4A, Algorithm A4). Since each node on the rule structure graph has a NodeSide attribute (reactant/product/both), this is translated as a relationship between the matched atom at that node and the rule itself (reactant/product/context). These relationships are represented as binary labels on bipartite edges between the rule and the atoms, resulting in the network graph called the **atom-rule graph** (Fig F4A, Algorithm A4), or AR graph. To visualize AR graphs (Rendering Conventions C3), we apply distinct visual attributes to the two node types (atom, rule) and the three edge types (reactant, product, context). Individual rule AR graphs are merged and processed to build the **model AR graph** (Fig F4B, Algorithm A5). The rules in a model draw from the same finite pool of structure types and binding interactions, so the model AR graph grows primarily with the number of rules in the model.


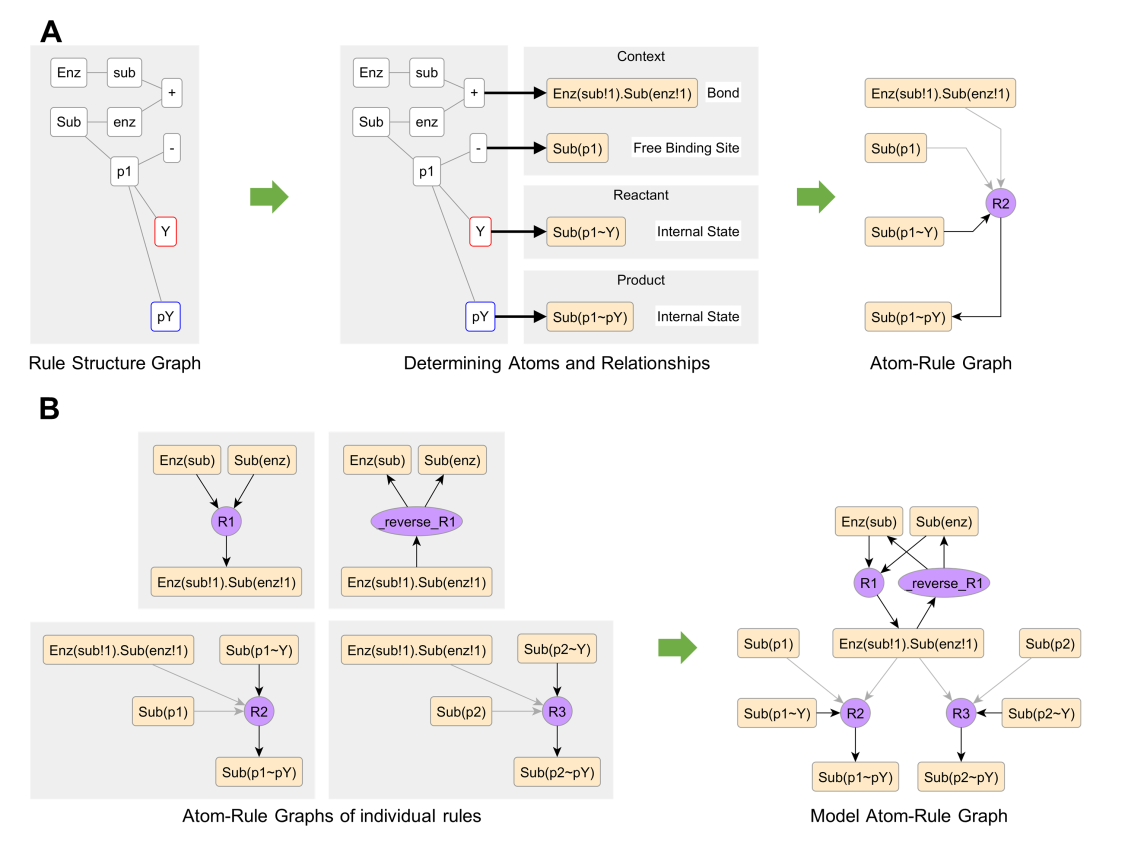


**Fig F4. Atom-Rule (AR) Graph. (A)** An atom represents a distinct type of modifiable structure: a free binding site, a bond, an internal state or a molecule type. It has a name specified using pattern syntax (brackets = containership, !{tag}=bond, ~{label}=internal state). Atoms are identified by examining source nodes on the rule structure graph (bold arrows). The atom-rule (AR) graph is a network graph with edges between atoms and a node labeled with the rule name. The NodeSide attribute of the source node (border color red=left, blue=right, gray=both) determines edge attributes on the AR graph (reactant, product and/or context). Context edges are rendered with a light color and reactant/product edges have a dark color. **(B)** Aggregating AR graphs of individual rules results in the model AR graph.

**Visualizing Function**: To build compact pathway diagrams, the elements of the model AR graph have to be organized and simplified. This involves four steps: removing background nodes, grouping atoms, grouping rules and compressing groups. Background removal consists of removing atoms and rules that are redundant for model comprehension (Fig. F5B, Algorithm A6). This greatly reduces the size of the graph. Grouping atoms involves identifying similarity between sites in the system, e.g., phospho-sites on the same molecule or bonds between the same pair of molecules (Fig F5C). Grouping rules requires computing similarity between rules based on their edge signatures, i.e., based on how each rule interacts with groups of atoms (Fig F5D, Algorithms A7 and A8). Compressing groups involves replacing groups of nodes by a representative group node and combining edges incident on individual members of the group and remapping them to the representative group node (Fig F5E, Algorithm A9). Background removal and atom grouping are performed using default heuristics whose choices can be modified with user input, then rule grouping is performed automatically based on those settings. The edge signature can be strict or permissive, i.e. it considers all three edge types or it considers only reactant and product edges and ignores context edges respectively. Using a permissive edge signature results in identifying a broader grouping of rules.

**
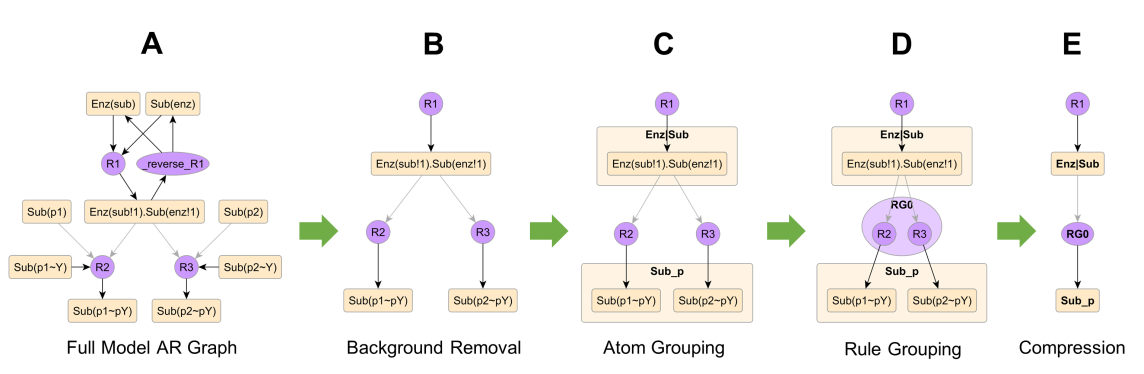
**

**Fig F5. Complexity reduction approaches. (A)** The full model AR graph from Fig F4. **(B)** Nodes redundant for human comprehension are tagged as background and removed. Here, that includes free binding sites and the unbinding rule. **(C)** Atoms are classified into groups. Here, the groups used are Enz|Sub to denote the enzyme-substrate bond and Sub_p to denote the phosphorylated sites. Steps (B) and (C) can be performed by a heuristic that can be modified with user annotation. **(D)** Rules are classified into groups by an automated comparison of their edge signatures. Here, rules R2 and R3 have similar edge signatures: a context edge from Enz|Sub and a product edge into Sub_p, so they are grouped under the same label RG0. **(E)** Each group of nodes is replaced by a single representative node and edges incident on individual members of the group are merged together.

**Algorithm Time Complexity**: If *p* represents the size of a pattern, building a pattern structure graph from the pattern syntax is *O(p)* (Algorithm A1). If *m_L_, m_R_* represent the size of patterns on left and right sides of a rule respectively, building a correspondence map between reactants and products before merging is *O(m_L_*m_R_)* (Algorithm A2). Building the rule structure graph from the correspondence map (Algorithm A3) and building the atom-rule graph from the rule structure graph (Algorithm A4) are both *O(m)* where *m* represents the size of the rule. Since rules are typically finite and bounded in size (Fig F6), the contribution of rule size can be assumed to be *O(1)*. So, building the AR graph (Algorithm A5), removing background (Algorithm A6), identifying rule groups using edge signatures (Algorithms A7 and A8) and collapsing group nodes (Algorithms A9), are all *O(n)*, where *n* is the number of rules.


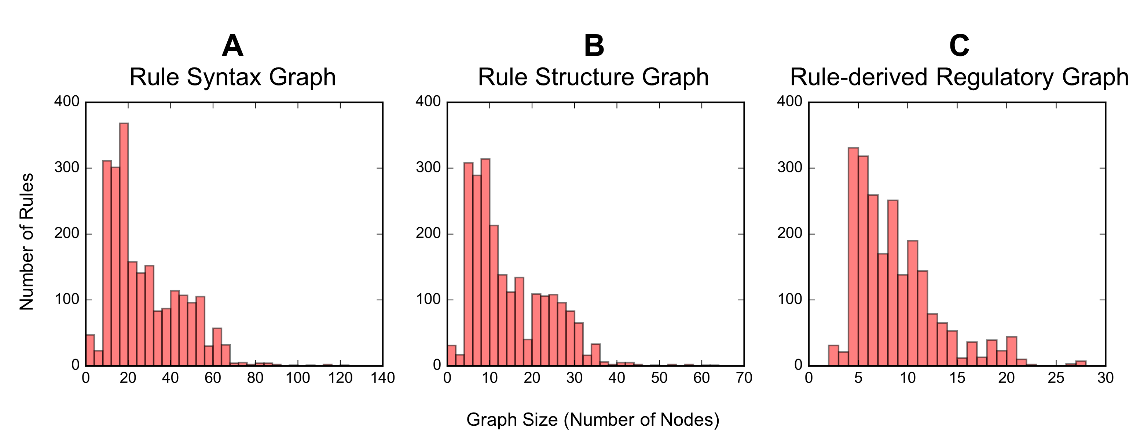


**C**

**BA**

**A**

**Fig F6. Rule size distributions.** Distribution of rule sizes for 2239 rules from 27 rule-based models in the literature models when represented as **(A)** rule syntax graph, **(B)** rule structure graph and **(C)** atom-rule graph for each rule. In all three representations, it is seen that large rules are infrequently encountered.

**Heuristics for Background Removal:** The default heuristic makes the following choices for including or excluding atoms/rules from the background

1. All free binding sites are included in background. All bonds are excluded.
2. When internal states are encountered on a component, the first such internal state is included in background. Typically, this would assign default states such as unphosphorylated state to the background.
3. All unidirectional rules are excluded from background.
4. For bidirectional rules, the forward rule is excluded from background, but the reverse rule is included.
5. Molecule atoms associated with molecule creation/deletion rules are excluded from background.

**Heuristics for Grouping Atoms:** The default heuristic for atom grouping makes the following choices:

1. Bonds are grouped based on the molecules they link, e.g., X(a!1).Y(b!1) is placed in the group X|Y.
2. Internal states are grouped based on the molecule they are present in and the states they represent, e.g., X(a~p) is placed in the group X_p.

**References**

[1] J. S. Hogg, L. A. Harris, L. J. Stover, N. S. Nair, and J. R. Faeder, “Exact hybrid particle/population simulation of rule-based models of biochemical systems.,” *PLoS Comput. Biol.*, vol. 10, no. 4, p. e1003544, Apr. 2014.

[2] N. W. Lemons, B. Hu, and W. S. Hlavacek, “Hierarchical graphs for rule-based modeling of biochemical systems.,” *BMC Bioinformatics*, vol. 12, p. 45, 2011.

[3] C.-F. Tiger, F. Krause, G. Cedersund, R. Palmér, E. Klipp, S. Hohmann, H. Kitano, and M. Krantz, “A framework for mapping, visualisation and automatic model creation of signal-transduction networks.,” *Mol. Syst. Biol.*, vol. 8, no. 578, p. 578, Jan. 2012.

[4] J. A. Sekar and J. R. Faeder, “Rule-based modeling of signal transduction: a primer.,” *Methods Mol. Biol.*, vol. 880, pp. 139–218, 2012.

[5] J. R. Faeder, M. L. Blinov, and W. S. Hlavacek, “Rule-based modeling of biochemical systems with BioNetGen,” *Methods Mol. Biol.*, vol. 500, no. 1, pp. 113–167, 2009.

[6] M. Blinov, J. Yang, J. R. Faeder, and W. S. Hlavacek, “Graph theory for rule-based modeling of biochemical networks,” *Trans. Comput. Syst. Biol. VII*, vol. 4230, pp. 89–106, 2006.

S1.2: Algorithms

**A1 Pattern Structure Graph**

**Goal:** Given a BioNetGen pattern, compose a graph in which each element of the pattern is a node and each edge is a hierarchical relationship. The node has attributes (NodeIndex, NodeLabel, NodeType) where NodeLabel is a name, NodeType is one of {molecule, component, internal state, bond state} and NodeIndex is a unique index. Bond state nodes have NodeLabel + to indicate bonds and – to indicate unbound state.

**Example:** Given the pattern A(b!1).B(a~x!1), we get a graph with six nodes { (1,A,molecule), (2,B,molecule), (3,b,component), (4,a,component), (5,x,internal state), (6,+,bond state) } and five edges {(1,3),(2,4),(2,5),(3,6),(4,6)}.

**Input:** Pattern $p=(x)$ and a map $type:p\to\{$molecule, component, internal state, bond}. Each element $x$ in $p$ has one of the following forms:

$\left( m,m_{t} \right),$ is a molecule with index $m$ of type $m_{t}$,

$\left( c,c_{t},m \right)$, a component with index $c$ of type $c_{t}$ in molecule $m$,

$\left( s,s_{t},c \right),$ is an internal state with index $s$ of type $s_{t}$ on component $c$,

$(b,c_{1},c_{2})$ is a bond with index $b$ between components $c_{1}$ and $c_{2}$,

$(b,c)$ is a wildcard bond with index $b$ on component $c$

| **1 2**  **3**  **4**  **5 6 7 8 9 10 11 12 13 14 15 16 17 18 19 20**  **21**  **22**  **23** | initialize empty sets $V,E$ and function $bonded$  for each $x$ in $p$,  if $x=(m,m_{t})$ and $type\left( x \right)=$ molecule  add node ($m$,$m_{t}$,molecule) to $V$  if $x=(c,c_{t},m)$ and $type\left( x \right)=$ component  add node ($c, c_{t}$, component) to $V$  add edg e $\left( m,c \right)$ to $E$  $bonded\left( c \right)\leftarrow$ False  if $x=\left( s,s_{t},c \right)$ and $type\left( x \right)=$ internal state  add node ($s$,$s_{t}$,internal state) to $V$  add edge $(c,s)$ to $E$  if $x=(b,c_{1},c_{2})$ and $type\left( x \right)=$ bond  add node ($b$,$+$, bond state) to $V$  add edges $\left( c_{1},b \right),(c_{2},b)$ to $E$  $bonded\left( c \right)\leftarrow$ True  if $x=\left( b,c \right)$  add node ($b$,$+$,bond state) to $V$  add edge $(c,b)$ to $E$  $bonded\left( c \right)\leftarrow$True  for each component $x$ in $p$ such that $bonded\left( c \right)=$False  add node ($u$,$-$,bond state) to $V$, where $u$ is a unique id  add edge $\left( c,u \right)$ to $E$  $G\leftarrow\left( V,E \right)$ |
| --- | --- |

**Output:** Pattern structure graph $G$.

**Complexity:** $\mathcal{O}\left( \left| p \right| \right)$ , i.e. linear in the size of the pattern.

**Notes:** From above, $\left| E \right|\leq2\left| V \right|$. We will treat $\left| E \right|\propto\left| V \right|$ for pattern structure graphs in A2 and A3.

**A2 Correspondence Map**

**Goal:** Given a BioNetGen rule, synthesize a partial map between reactants and products.

**Example:** Given a rule A(b~x) -> A(b~y), the reactant structure graph is {(1,A,molecule), (2,b,component), (3,x,internal state), (4,-,bond state)}. Similarly, the product structure graph is {(5,A,molecule), (6,b,component), (7,y,internal state), (4,-,bond state)}. The correspondence map is {(1->5), (2->6), (4->8)}. For simplicity, we can denote this as A->A, b->b.

**Input:** Rule $\left( p_{l},p_{r} \right)$ where $p_{l}:=\left( x_{l} \right)$, $p_{r}:=(x_{r})$ and $type:\left\{ x | x\in p_{l}\cup p_{r} \right\}\to${ molecule, component, internal state, bond }. $p_{l}$ and $p_{r}$ are merged patterns of the left and right sides of the rule respectively.

| **1** | $M^{l}=\left( x \right)$, a sequence with molecules $x$ drawn from $p_{l}$ |
| --- | --- |
| **2** | $M^{r}=\left( x \right)$, a sequence with molecules $x$ drawn from $p_{r}$ |
| **3** | for each $x\in M^{l}$ such that $x=(m,m_{t})$ |
| **4** | $C_{x}=\left( y \right)$, a sequence with components $y$ drawn from $p_{l}$ s.t. $y=(c,c_{t},m)$ |
| **5** | for each $x\in M^{r}$such that $x=(m,m_{t})$ |
| **6** | $C_{x}=\left( y \right)$, a sequence with components $y$ drawn from $p_{r}$ s.t. $y=(c,c_{t},x)$ |
| **7** | initialize empty sets $dom$, $img$ and function $\psi:dom\to img$ |
| **8** | for each $x\in M^{l}$ such that $x=(m^{x},m_{t}^{x})$ and $x$ not in $dom$ |
| **9** | for each $y\in M^{r}$such that $x=(m^{y},m_{t}^{y})$ and $m_{t}^{x}=m_{t}^{y}$ and $y$not in $img$ |
| **10** | if exists $C_{x}\to C_{y}$ preserving component type, presence of internal state, wildcard |
| **11** | add $x$ to $dom$ and $y$ to $img$ |
| **12** | $\psi\left( x \right)\leftarrow y$ |
| **13** | for each molecule map $(x\to y)$ in $\psi$ |
| **14** | for each $a$ in $C_{x}$ such that $a=\left( c^{a},c_{t}^{a},m^{a} \right)$and $a$ not in $dom$ |
| **15** | for each $b$ in $C_{x}$ such that $b=\left( c^{b},c_{t}^{b},m^{b} \right)$and $c_{t}^{a}=c_{t}^{b}$ and $b$ not in $img$ |
| **16** | add $a$ to $dom$ and $b$ to $img$ |
| **17** | $\psi\left( a \right)\leftarrow b$ |
| **18** | for each component map $\left( a\to b \right)$ in $\psi$ such that $a=\left( c^{a},c_{t}^{a},m^{a} \right),b=\left( c^{b},c_{t}^{b},m^{b} \right)$ |
| **19** | if exists $(s^{l},s_{t}^{l},c^{l})\in p^{l}, (s^{r},s_{t}^{r},c^{r})\in p^{r}$ such that $c^{l}=c^{a},c^{r}=c^{b},s_{t}^{l}=s_{t}^{r}$ |
| **20** | add $(s^{l},s_{t}^{l},c^{l})$ to $dom$ and $(s^{r},s_{t}^{r},c^{r})$ to $img$ |
| **21** | $\psi\left( \left( s^{l},s_{t}^{l},c^{l} \right) \right)\leftarrow(s^{r},s_{t}^{r},c^{r})$ |
| **22** | if exists $(b^{l},c^{l})\in p^{l}, (b^{r},c^{r})\in p^{r}$ such that $c^{l}=c^{a},c^{r}=c^{b}$ |
| **23** | add $\left( b^{l},c^{l} \right)$ to $dom$ and $\left( b^{r},c^{r} \right)$ to $img$ |
| **24** | $\psi\left( \left( b^{l},c^{l} \right) \right)\leftarrow\left( b^{r},c^{r} \right)$ |
| **25** | for each bond $g\in p_{l}$ such that $g=\left( b^{l},c_{1}^{l},c_{2}^{l} \right)$ and $g$ not in $dom$ |
| **26** | for each bond $h\in p_{r}$ such that $h=\left( b^{r},c_{1}^{r},c_{2}^{r} \right)$ and $h$ not in $img$ |
| **27** | if exists $\left\{ c_{1}^{l},c_{2}^{l} \right\}\to\left\{ c_{1}^{r},c_{2}^{r} \right\}$ in $\psi$ |
| **28** | add $g$ to $dom$ and $h$ to $img$ |
| **29** | $\psi\left( g \right)\leftarrow h$ |

**Output:** Correspondence map $\psi:p_{l}↛p_{r}$, where $↛$ indicates that it is a partial map.

**Complexity:** $\mathcal{O}\left( \left| p_{l} \right|*\left| p_{r} \right| \right)$. This can be considered $\mathcal{O}\left( 1 \right)$as large rule sizes are rare (Fig F6A).

**Notes:** Since the pattern structure graph has one node for every element in a pattern, the correspondence map can also be defined equivalently on pattern structure graphs, i.e. $\psi:V^{l}↛V^{r}$

**A3 Rule Structure Graph**

**Goal:** Given a BioNetGen rule and a correspondence map between reactants and products, synthesize a graph in which the left and right sides are merged together. In addition to NodeIndex, NodeLabel, and NodeType, use the NodeSide attribute to indicate which side of the rule each node is derived from.

**Example:** Given the rule A(b~x) -> A(b~y) and the map A->A, b->b, it produces a graph with five nodes {(1,A,molecule,both), (2,b,component,both), (3,x,internal state,left), (4,y,internal state,right), (5,-,bond state,both)} and four edges {(1,2), (2,3), (2,4), (2,5)}. The nodes with NodeSide ‘both’ have been merged from both sides of the rule whereas the nodes with NodeSide equals ‘left’ or ‘right’ come uniquely from the reactant or product sides of the rule respectively.

**Input:** Rule $\left( G^{l},G^{r},\psi\right)$ where $G^{l}:=\left( V^{l},E^{l} \right), G^{r}:=\left( V^{r},E^{r} \right)$are merged pattern structure graphs and $\psi:V^{l}↛V^{r}$ is a correspondence map.

| **1**  **2**  **3**  **4**  **5**  **6**  **7**  **8**  **9**  **10**  **11**  **12**  **13**  **14**  **15** | initialize empty sets $V,E$  for each node $v$ in $V^{l}$, label NodeSide($v$)=left  for each node $v$ in $V^{r}$, label NodeSide($v$)=right  for each map $v\to v'$ in $\psi$,  label NodeSide($v$) = NodeSide($v’$) = both  for each edge $\left( v,v' \right)$ in $E^{l}$, add edge $\left( v,v' \right)$ to $E$  for each edge $\left( v,v' \right)$ in $E^{r}$  if NodeSide($v$)=NodeSide($v^{'}$)=both  next edge  if NodeSide($v$)=both  $v\leftarrow\psi^{-1}(v)$  if NodeSide($v'$)=both  $v^{'}\leftarrow\psi^{-1}\left( v^{'} \right)$  add edge $\left( v,v^{'} \right)$ to $E$  $G\leftarrow\left( V,E \right)$ |
| --- | --- |

**Output:** Rule structure graph $G$

**Complexity:** $\mathcal{O}\left( \left| V^{l} \right|+\left| V^{r} \right|+\left| E^{l} \right|+\left| E^{r} \right| \right)\mathcal{\approx O}\left( \left| V^{l} \right|+\left| V^{r} \right| \right)$ since $\left| E \right|\propto\left| V \right|$for pattern structure graphs. The complexity is linear in the size of the rule $\left| V^{l} \right|+\left| V^{r} \right|$. This can be considered $\mathcal{O}\left( 1 \right)$ as large rule sizes are rare (Fig F6A).

**Notes:** The rule structure graph has the same property of the pattern structure graph, i.e. $\left| E \right|\propto\left| V \right|$ .

**A4 Atom-Rule Graph**

**Goal:** The atom-rule (AR) graph is a network graph in which nodes have attributes NodeType and NodeLabel and edges have attributes Reactant, Product and Context respectively which take binary values 0/1 each. NodeType can be atom or rule and NodeLabel is sufficient to index nodes uniquely. Given a rule structure graph of a rule, synthesize an AR graph in which one node is labeled with the name of the rule.

**Example:** Given the rule labeled rule1 of the form A(b~x) -> A(b~y) and its corresponding rule structure graph (see A3 Example), build a network graph with four nodes having NodeLabel equals rule1, A(b), A(b~x), A(b~y) respectively. The node labeled rule1 is of NodeType rule and the other nodes are of node type atom. Draw edges { ( rule1,A(b) ), (rule1, A(b~x) ), (rule1, A(b~y) ) } respectively with edge labels 001, 100, 010 respectively, where 001 indicates that edge attributes are Reactant=0,Product=0,Context=1 respectively. Each node and edge is drawn by examining a corresponding node on the rule structure graph and its neighbors.

**Input:** Rule structure graph $G^{s}:=\left( V^{s},E^{s},r \right)$ where $r$ is a label indexing the rule.

| **1**  **2**  **3**  **4**  **5**  **6**  **7**  **8**  **9**  **10**  **11**  **12**  **13**  **14**  **15**  **16**  **17**  **18** | for each node $v$ in $V^{s}$, initialize empty set Parent($v$)  for each edge $\left( v,v^{'} \right)$ in $E^{s}$  if $v$ is a molecule with NodeLabel $m_{t}$  if $v'$ is a component  add label $"m_{t}"$ to Parent($v$’)  if $v$ is a component with NodeLabel $c_{t}$ and parent $"m_{t}"$  if $v'$ is an internal or bond state  add label $"\text{m}_{\text{t}}\left( \text{c}_{\text{t}} \right)"$ to Parent($v$’)  initialize empty sets $V,E$  add node $v_{r}$=($r$,rule) to $V$  for each node $v^{s}$ in $V^{s}$  determine atom NodeLabel $a$ from Table below  make node $v_{a}=$($a$, atom),  if $v_{a}$ not in $V$,  add node $v_{a}$ to $V$  add edge $(v_{a},v_{r})$ to $E$ with default labels (Reactant=0,Product=0,Context=0)  apply label modification of edge $(v_{a},v_{r})$ from Table  $G\leftarrow\left( V,E \right)$ |
| --- | --- |

**Output:** Atom-rule graph $G$.

**Complexity:** $\mathcal{O}\left( \left| V^{s} \right|+\left| E^{s} \right| \right)\mathcal{\approx O}\left( \left| V^{s} \right| \right)$ since $\left| E \right|\propto\left| V \right|$for the rule structure graph. In other words, the complexity is linear in the size of the rule structure graph $\left| V^{S} \right|$. This can be considered $\mathcal{O}\left( 1 \right)$ as large rule structure graph sizes are rare (Fig F6B).

**Notes:** From above, $\left| E \right|=\left| V \right|-1$ for the atom-rule graph of an individual rule. Since rule sizes are bounded, atom-rule graph sizes are bounded also (Fig F6C). So we treat$\left| E \right|\approx\left| V \right|\approx$constant for the individual atom-rule graphs in A5.

| Attributes of node $v^{s}$ on rule structure graph of rule labeled $r$ | | | | NodeLabel $a$ for atom node $v_{a}$ | Label modification for edge $\left( v_{a},v_{r} \right)$ |
| --- | --- | --- | --- | --- | --- |
| NodeType | NodeLabel | NodeSide | Parents |  |  |
| Molecule | $m_{t}$ | Left | None | $m_{t}$ | Reactant$\leftarrow$ 1 |
|  |  | Right |  |  | Product $\leftarrow$ 1 |
|  |  | Both |  | - | - |
| Internal State | $s_{t}$ | Left | $m_{t}(c_{t})$ | $m_{t}\left( c_{t}\sim s_{t} \right)$ | Reactant $\leftarrow$ 1 |
|  |  | Right |  |  | Product $\leftarrow$ 1 |
|  |  | Both |  |  | Context$\leftarrow$ 1 |
| Bond State | $+$ | Left | $m_{t}\left( c_{t} \right),m_{t}^{'}\left( c_{t}^{'} \right)$ | $m_{t}\left( c_{t}!1 \right).m_{t}^{'}\left( c_{t}^{'}!1 \right)$ | Reactant$\leftarrow$ 1 |
|  |  | Right |  |  | Product$\leftarrow$ 1 |
|  |  | Both |  |  | Context$\leftarrow$1 |
|  |  | Both | $m_{t}\left( c_{t} \right)$ | $m_{t}\left( c_{t}!+ \right)$ | Context $\leftarrow$1 |
|  | $-$ | Left | $m_{t}\left( c_{t} \right)$ | $m_{t}\left( c_{t} \right)$ | Reactant$\leftarrow$ 1 |
|  |  | Right |  |  | Product $\leftarrow$1 |
|  |  | Both |  |  | Context $\leftarrow$ 1 |

**A5 Model AR Graph**

**Goal:** Given a set of rule atom-rule graphs of individual rules, merge them into a single graph. Additionally, remap wildcard bonds (e.g. A(b!+)) to matching fully specified bonds (e.g. A(b!1).B(a!1)).

**Example:** Given two graphs with nodes {rule1, atom1, atom2} and {rule2, atom2, atom3} respectively, the resultant graph will have the nodes {rule1, rule2, atom1, atom2, atom3}. Edges on the resultant graph have all the labels of the corresponding edges on the individual graphs.

**Input:** Set of atom-rule graphs $\left\{ G_{r}|G_{r}:=\left( V_{r},E_{r} \right) \right\}$, where $r$ indexes the rule.

| **1** | $V\leftarrow\bigcup_{\forall r} V_{r}, E\leftarrow\bigcup_{\forall r} E_{r}$ |
| --- | --- |
| **2**  **3**  **4**  **5**  **6**  **7**  **8**  **9** | edge label conflicts are resolved using Boolean OR.  for each wildcard $w$ in $V$  for each edge $\left( w,x \right)$ in $E$,  for each bond $b$ in $V$ such that $w$ matches $b$  add edge $\left( b,x \right)$ to $E$ with same labels as $\left( w,x \right)$  delete edge $\left( w,x \right)$ in $E$  delete node $w$ in $V$ $G\leftarrow\left( V,E \right)$ |

**Output:** model AR graph $G$.

**Complexity:** $\mathcal{O}\left( \Sigma_{r}\left( \left| V_{r} \right|+\left| E_{r} \right| \right)+\left| w \right|*\left| b \right| \right)$ where $V_{r},E_{r}$ are nodes and edges of AR graphs of individual rules indexed by $r$, $|w|$ and $\left| b \right|$ are number of wildcards and bonds respectively. Since $\left| V_{r} \right|\approx\left| E_{r} \right|\approx$constant for individual atom-rule graphs (A4 Notes) and wildcards are rarely used, the average complexity is $\mathcal{O}\left( n \right)$, where $n$is the number of rules.

**Notes:** Since there are only two node types, the model AR graph is also represented as the tuple $\left( V_{A},V_{R},E \right)$ where $V_{A}$ and $V_{R}$ partition $V$ into atoms and rules respectively. Since $\left| E \right|\approx$ constant for AR graphs of individual rules, we treat $\left| E \right|\propto\left| V_{R} \right|$ for the model AR graph in A6 and A7.

**A6 Background Removal**

**Goal:** Given a model AR graph, remove background nodes.

**Example:** Given a graph {rule1, rule2, atom1, atom2}, if the set of background nodes are {rule2, atom1}, then the output graph has the nodes {rule1, atom2}. Edges between non-background nodes are transferred as is to the new graph.

**Input:** Model AR graph $G:=\left( V,E \right)$, background assignment $Bkg:V\to\{0,1\}$

| **1**  **2**  **3** | $V^{'}\leftarrow V-\left\{ v \vert Bkg\left( v \right)=1,v\in V \right\}$  $E^{'}\leftarrow E-\left\{ \left( v,v^{'},EdgeLabel \right) \vert Bkg\left( v \right)+Bkg\left( v^{'} \right)\geq1,\left( v,v^{'},EdgeLabel \right)\in E \right\}$  $G'\leftarrow\left( V',E' \right)$ |
| --- | --- |

**Output:** model AR graph $G'$

**Complexity:** $\mathcal{O}\left( \left| V \right|+\left| E \right| \right)$, i.e. linear in the size of the model AR graph.

**A7 Edge Signature of a Rule**

**Goals:** For a particular rule node on the model AR graph, compute a signature from adjacent nodes, edges and edge attributes.

**Example:** Given a graph {rule1, atom1, atom2, atom3} with edges { (rule1,atom1,100), (rule1,atom2,010) , (rule1,atom3,001) }, where edge label 100 indicates edge attributes Reactant=1,Product=0,Context=0, the edge signature for rule1 is (atom1):(atom2):(atom3). Suppose a partial grouping function is provided on patterns, say atom1->atomgroup1 and atom2->atomgroup2, then the edge signature is (atomgroup1):(atomgroup2):(patt3).

**input**, Rule $v_{r}\in V_{R}$. model AR graph $G_{M}:=\left( V_{A},V_{R},E_{M} \right)$, partial grouping function on atoms
 $f:V_{A}↛Labels$.

| **1**  **2**  **3**  **4**  **5**  **6**  **7**  **8**  **9**  **10**  **11** | initialize empty sets $Re, Pr, Co$  for each edge $(v_{a},v_{r},EdgeLabel)$ in $E_{M}$  string $S\leftarrow v_{a}$ if $v_{a}$ not in domain of $f$  string $S\leftarrow f(v_{a})$ if $v_{a}$ in domain of $f$  add $S$ to $Re$ if Reactant=1 in $EdgeLabel$  add $S$ to $Pr$ if Product=1 in $EdgeLabel$  add $S$ to $Co$ if Context=1 in $EdgeLabel$  string $S_{1}\leftarrow$ sorted and concatenated elements of $Re$  string $S_{2}\leftarrow$ sorted and concatenated elements of $Pr$  string $S_{3}\leftarrow$ sorted and concatenated elements of $Co$  string $Sgn\left( v_{r} \right)\leftarrow$ $S_{1}:S_{2}:S_{3}$ |
| --- | --- |

**Output:** Edge signature $Sgn\left( v_{r} \right)$

**Complexity:** $\mathcal{O}\left( \left( \frac{\left| E_{M} \right|}{\left| V_{R} \right|} \right)^{2} \right)\mathcal{\approx O}\left( 1 \right)$, since $\left| E_{M} \right|\propto\left| V_{R} \right|$ on the model AR graph (A5 Notes).

**Notes** A more permissive edge signature can be obtained by discounting context edges, i.e. $Sgn\left( v_{r} \right)\leftarrow$ concatenated $S_{1}:S_{2}$ only.

**A8: Grouping Atoms and Rules**

**Goal:** Given a model AR graph and an optional atom grouping, group rules according to edge signature.

**Example** Given a graph {rule1, rule2, atom1, atom2} with edges { (rule1,atom1,product), (rule2,atom2,product) } and atomgroup1={atom1,atom2}, assign rule1 and rule2 to the same group rulegroup1.

**Input:** Model AR graph $G_{M}:=\left( V_{A},V_{R},E_{M} \right)$, partial group assignment $GrpA:V_{A}↛Labels$ for atoms.

| **1**  **2**  **3**  **4**  **5**  **6**  **7**  **8**  **9** | for each node $v_{r}$ in $V_{R}$  $Sgn\left( v_{r} \right)\leftarrow$ Edge signature of $v_{r}$ given function $GrpA$ using S7.6  if $Sgn(v_{r})$ not in domain of $count$  $count\left( Sgn\left( v_{r} \right) \right)\leftarrow0$  increment $count(Sgn(v_{r}))$  if $count\left( Sgn\left( v_{r} \right) \right)=2$  $ind\left( Sgn\left( v_{r} \right) \right)\leftarrow x$, where $x$ is a unique label  for each node $v_{r}$ in $V_{R}$ such that $count\left( Sgn\left( v_{r} \right) \right)>1$  $GrpR\left( v_{R} \right)\leftarrow ind\left( Sgn\left( v_{r} \right) \right)$ |
| --- | --- |

**Output:** partial group assignment $GrpR:V_{R}↛Labels$ for rules.

**Complexity:** $\mathcal{O}\left( \left| V_{R} \right| \right)$, i.e. linear in the number of rules.

**Notes:** If the edge signature is built only from reactant and product edges and does not use context edges, then the grouping is more permissive and fewer groups are obtained with larger group sizes.

**A9: Collapsing Groups**

**Goal:** Given a model AR graph with groups, replace each group of nodes by a single node labeled with the group name.

**Example:** Given a graph with nodes {atom1,atom2,atom3,rule1,rule2,rule3} in which atomgroup1 = {atom1, atom2}, rulegroup1 = {rule1, rule2}, the new graph has nodes {atomgroup1, atom3, rulegroup1, rule3}. Edges incident on either atom1 or atom2 are remapped to atomgroup1 and edges incident on rule1 or rule2 are remapped to rulegroup1.

**Input:** Model AR graph $G_{M}:=\left( V_{A},V_{R},E_{M} \right)$, partial group assignments $GrpA,GrpR$ on $V_{A}, V_{R}$ respectively.

| **1**  **2**  **3**  **4**  **5**  **6**  **7**  **8** | initialize empty sets $V,E$ and function $Remap$  for each node $v$ in $V_{A}\cup V_{R}$  make node $x$ from $v$ according to Table below  add node $x$ to $V$  $Remap\left( v \right)\leftarrow x$  for each edge $\left( v_{a},v_{r},EdgeLabel \right)$ in $E_{M}$  add edge $\left( Remap\left( v_{a} \right),Remap\left( v_{r} \right),EdgeLabel \right)$ to $E$  $G\leftarrow\left( V,E \right)$ |
| --- | --- |

**Output:** merged AR graph $G$.

**Complexity:** $\mathcal{O}\left( \left| V_{A} \right|+\left| V_{R} \right|+\left| E_{M} \right| \right)\mathcal{\approx O}\left( \left| V_{A} \right|+\left| V_{R} \right| \right)$, since $\left| E_{M} \right|\propto|V_{R}|$, i.e. linear in the size of the model AR graph $\left| V_{A} \right|+\left| V_{R} \right|$.

| NodeType of $v$ | $v\in$domain of $GrpA$ | $v\in$domain of $GrpR$ | $x=$(NodeLabel,NodeType,isGroup) |
| --- | --- | --- | --- |
| atom | True | - | ($GrpA\left( v \right)$, atom, True) |
|  | False | - | ($v$, atom, False) |
| rule | - | True | ($GrpR\left( v \right)$, rule, True) |
|  | - | False | ($v$, rule, False) |

S1.3: Rendering Conventions

**C1 Site Graph**

**Goal:** Given a pattern structure graph, draw a site graph by nesting components within molecules, internal states within components and drawing bonds as edges between components.

**Example:** Given a pattern A(b!1).B(a~x!1), nest b within A, a within B, x within a and add an edge between a and b.

**Input:** Pattern structure graph $G:=\left( V,E \right)$

| **1**  **2**  **3**  **4**  **5**  **6**  **7**  **8**  **9**  **10**  **11**  **12**  **13**  **14** | for each node $v$ in $V$  if type={molecule,component,internalstate} and name=S  render as node labeled S  for each node $v$ in $V$ such that type=component  if exists $\left( v,v^{'} \right)$ in $E$ such that  type=molecule for $v'$, then nest $v$ in $v'$  type=internalstate for $v'$, then nest $v'$ in $v$  for each node $v$ in $V$ such that type=bond  ignore if name=$-$  if name=$+$ and adjacent to two components $c,c'$  render as edge between renderings of $c,c'$  if name=$+$ and adjacent to only one component $c$  render as node labeled $+$  add edge to rendering of $c$ |
| --- | --- |

**Output:** Site graph

**C2 Compact Rule Visualization**

**Goal:** Given a rule structure graph, draw a site graph with the nodes labeled side=both and render nodes labeled side=left or side=right with special conventions.

**Example:** Given a rule A(b~x)-> A(b~y) and a rule structure graph {A,b,-,x,y} nest b within A, x within b, y within b, add ChangeState node, add directed edge from x to ChangeState, add directed edge from ChangeState to y.

**Input:** rule structure graph $G:=\left( V,E \right)$

| **1**  **2**  **3**  **4**  **5**  **6**  **7**  **8**  **9**  **10**  **11**  **12**  **13**  **14**  **15**  **16**  **17**  **18**  **19**  **20** | for each node $v$ in $V$  if side=both or type=internal state, render using site graph conventions  if type=molecule and side=left  add node labeled DeleteMol  add directed edge from $v$ to DeleteMol  if type=molecule and side=right  add node labeled AddMol  add directed edge from to AddMol to $v$  if type=bond and name=$+$ and side=left  replace with node labeled DeleteBond  add directed edges from adjacent components to DeleteBond  if type=bond and name=$+$ and side=right  replace with node labeled AddBond  add directed edges to adjacent components from AddBond  if type=component and exists two adjacent states $s,s'$  if side=left for $s$ and side=right for $s'$  add node labeled ChangeState  nest ChangeState node within $v$  add directed edge from $s$ to ChangeState  add directed edge from ChangeState to $s'$ |
| --- | --- |

**Output:** compact rule visualization

**C3 Atom-Rule Graphs**

**Goal:** Given an AR graph draw each node and edge according to provided conventions for atom and rule node types and reactant, product and context edge types. If a grouping scheme is provided, draw groups around the respective sets of nodes.

**Example:** Given graph {atom1, atom2, atom3, rule1, rule2, rule3} and groups atomgroup1={atom1, atom2}, rulegroup1={rule1,rule2}, draw nodes {atom1, atom2, atom3} using atom conventions, nodes {rule1, rule2, rule3} using rule node conventions, node atomgroup1 around {atom1, atom2} and node rulegroup1 around {rule1,rule2}.

**Input:** AR graph $G:=\left( V,E \right)$, two node rendering conventions for atom and rule respectively, three edge rendering conventions for reactant, product and context respectively, and optionally a partial group assignment $Grp:V\to Labels$.

| **1**  **2**  **3**  **4**  **5**  **6**  **7**  **8**  **9**  **10**  **11**  **12**  **13**  **14**  **15** | for each label $s$ in $img\left( Grp \right)$  draw node labeled $s$  for each node $v$ in $V$  if type=atom and name=S  render with atom node conventions and label S  if type=rule and name=S  render with rule node conventions and label S  if $Grp\left( v \right)=s$  nest within node labeled $s$  for each edge $\left( v,v^{'} \right)$ in $E$  if type of $v$ is atom and type of $v'$ is rule  if re=1, draw directed edge from $v$ to $v'$ with reactant edge conventions  if pr=1, draw directed edge from $v'$ to $v$ with product edge conventions  if co=1, draw directed edge from $v$ to $v'$ with context edge conventions  allow multiple edges between the same pair of nodes |
| --- | --- |

**Output:** Rendered AR graph
