# Supplementary material for: Automated visualization of rule-based models
Source: PLoS Comput Biol. 2017 Nov 13;13(11):e1005857. doi: 10.1371/journal.pcbi.1005857 (PMC5703574; doi:10.1371/journal.pcbi.1005857)
Supplement: S2 Appendix — (DOCX) [file pcbi.1005857.s007.docx]

S2 Appendix: Tutorial

# S2.0 Software Requirements

BioNetGen 2.3.0, available at <http://bionetgen.org/index.php/BioNetGen_Distributions>

yEd Graph Editor, available at <https://www.yworks.com/products/yed>

ensemble.bngl, available at *PLoS Comput. Biol. 2013* Supplementary Material*.*

# S2.1 Model Preprocessing

Consider the model ensemble.bngl from Suderman & Deeds (*PLoS Comput. Biol. 2013*)*.* Notice that the rules are not labeled. To get a model file ensemble_1.bngl with automatically labeled rules (from _R1 to _R272), we run ensemble.bngl with the action:

writeModel({format=>"bngl",suffix=>1})

Next we ensure that the patterns encoded in rules accurately reflect biological mechanisms. This involves correcting rules that were constructed in a non-canonical manner to accommodate simulation or syntax concerns. For example, in rules _R1 and _R2 below, the interaction of pheromone with Ste2 was modeled as if a new pheromone molecule is created during binding in _R1 and destroyed during dissociation in _R2. Presumably, this was done to simulate the effect of a constant free pheromone concentration.

_R1: Pheromone(ste2) + Ste2(pheromone) -> Pheromone(ste2!1).Ste2(pheromone!1) + Pheromone(ste2) _rateLaw1
_R2: Ste2(pheromone!1).Pheromone(ste2!1) -> Ste2(pheromone) _rateLaw2 DeleteMolecules

In ensemble_1.bngl, we modify them to be pure binding and dissociation rules:

_R1: Pheromone(ste2) + Ste2(pheromone) -> Pheromone(ste2!1).Ste2(pheromone!1) _rateLaw1
_R2: Ste2(pheromone!1).Pheromone(ste2!1) -> Ste2(pheromone)+Pheromone(ste2) _rateLaw2

Note also _R270 which models translocation of Ste5 from nucleus to cytosol.

_R270: Ste5(loc~n) -> Ste5(loc~c) Func1()

From the functions block, we see that Func1() = 0.3*(Gpa1_free/(Gpa1_free+2500)), where Gpa1_free is the observable Gpa1(ste4) from the observables block. The visualization tools in this work do not currently support dependencies encoded in functional rate laws such as the above. So we modify _R270 such that the effective kinetics remains the same and the rule has an additional pattern that encodes the dependence on Gpa1(ste4):

_R270: Ste5(loc~n) + Gpa1(ste4) -> Ste5(loc~c) + Gpa1(ste4) Func2()

where Func2() = 0.3/(Gpa1_free+2500). The modified model file ensemble_1.bngl with the older versions of rules commented out is attached with this document. For notes on observables, functions, and action commands, consult Sekar and Faeder (*Methods Mol. Biol. 2012*) and Faeder *et al.* (*Methods Mol. Biol. 2009*).

# S2.2 Individual Rule Visualization

To visualize individual rules, run ensemble_1.bngl with the following actions.

visualize({type=>"conventional",each=>1})
visualize({type=>"compact",each=>1})

The output of visualize() commands are typically files in .gml format (Graph Modeling Language), which is outlined at <http://docs.yworks.com/yfiles/doc/developers-guide/gml.html>. The each keyword ensures that the visualization constructed for each rule is kept in a separate file. The output files are named <model>_<visualization type>_<rule>.gml for rule visualizations and <model>_<visualization type>.gml for model visualizations respectively. The model files can be opened and laid out in yEd, a commercial graph editor freely available at <http://www.yworks.com/products/yed>.

# S2.3 Layout in yEd

yEd is a full-featured graph editor that provides several inbuilt algorithms for laying out parts or whole of the graph. It also enables laying out nodes and edges simultaneously or separately. A typical layout process involves multiple iterations of using inbuilt algorithms followed by manually moving around nodes and edges. The laid out diagram can be exported to formats such as png, jpeg, html, etc. Consider the compact rule visualization generated for rule _R247, i.e., ensemble_1_compact__R247.gml. On opening the file in yEd, it looks like this:


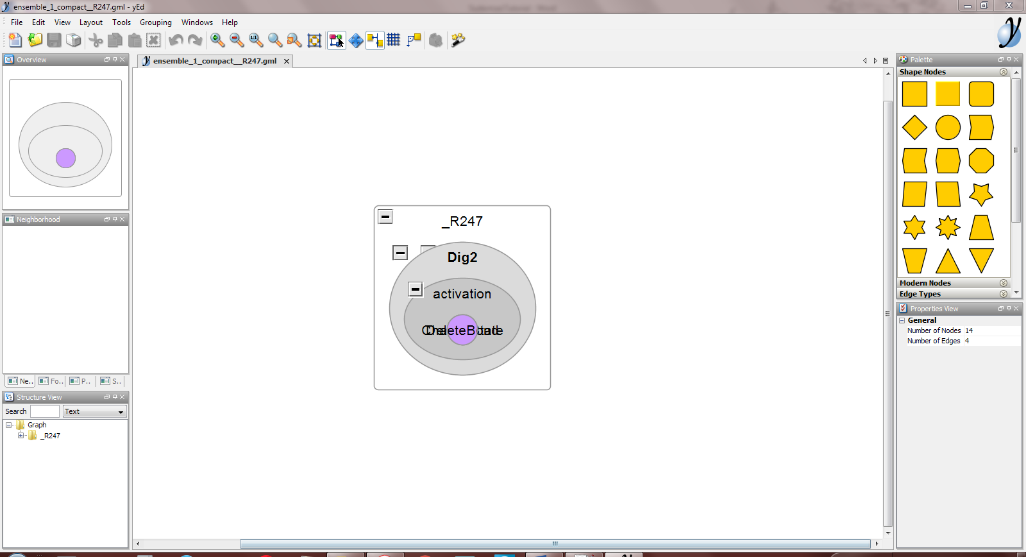


This is because visualize() generates the nodes and edges but not the layout, i.e., the positions of nodes and the individual line segments comprising the edges. So by default, all nodes have been placed at the center. Select Layout->One-Click Layout from the yEd menu. Now, it looks like this:


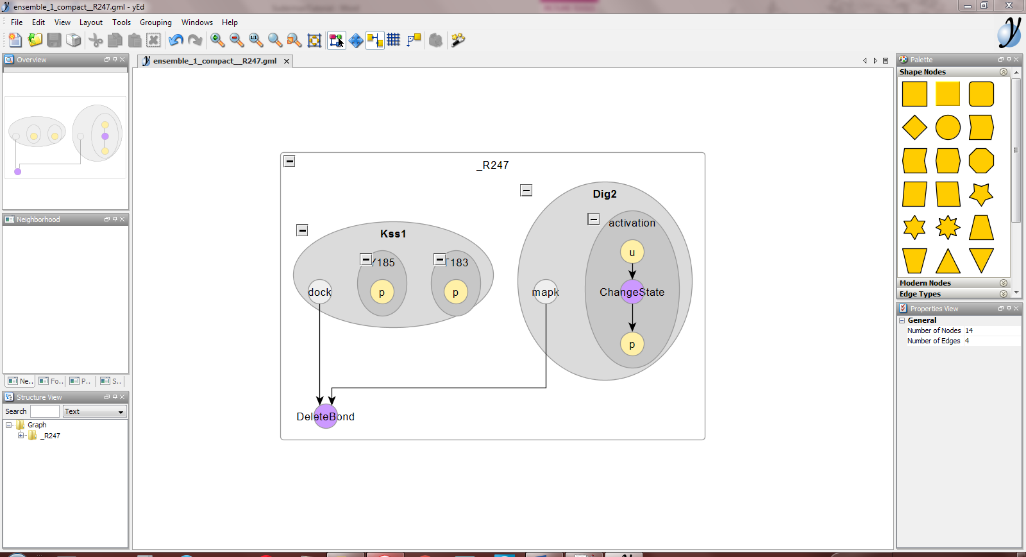


We provide the laid out ensemble_1_compact__R247.gml with this document.

# S2.4 Full Model Atom-Rule (AR) Graph

To visualize the full model atom-rule graph, run ensemble_1.bngl with the action

visualize({type=>"regulatory",background=>1,ruleNames=>1,suffix=>"full"})

The nodes removed in Step 1 of complexity reduction are called ‘background’, and the remaining nodes are called ‘foreground’. The background flag controls whether the background nodes must be displayed (1) or not (0, default). Since we wish to see the entire AR graph, we set its value to 1. The ruleNames parameter controls whether the labels of rule nodes are displayed (1) or not (0, default). Optionally, each=>1 can be used to output atom-rule graphs of individual rules. The command shown above generates the file ensemble_1_regulatory_full.gml. Import it into yEd. Since it has not been laid out yet, it looks like this:


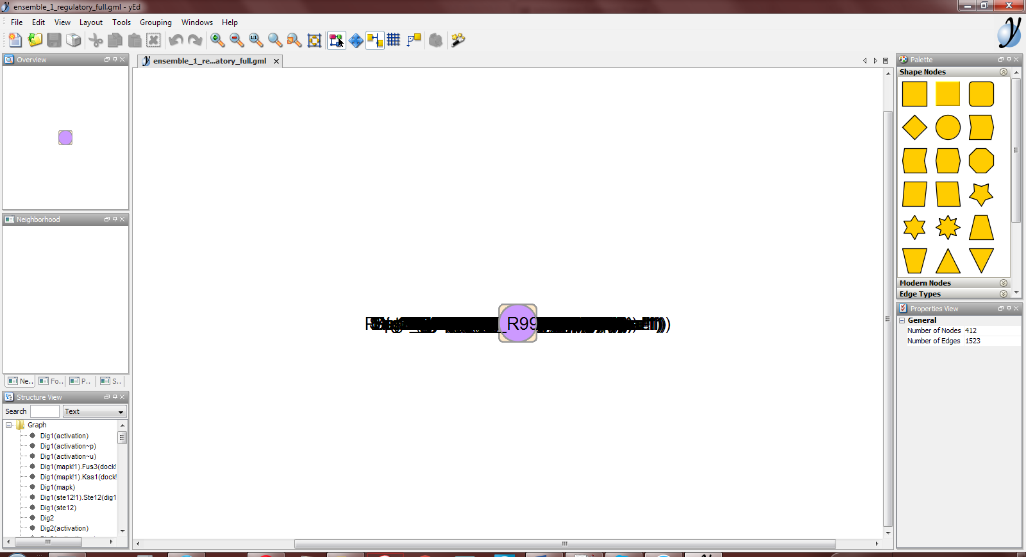


On the bottom left is the Structure View panel, which provides a list of nodes. To layout the graph, select Layout->Organic from the menu. Now it looks like this:


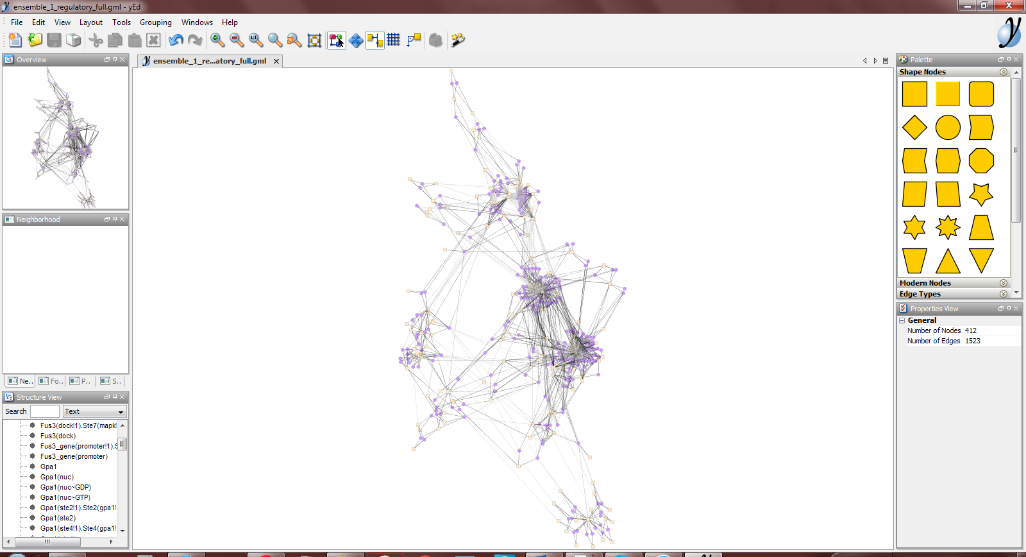


# S2.5 Default Template for Complexity Reduction

To generate the default choices for complexity reduction approaches in the form of a template file, run ensemble_1.bngl with the action

visualize({type=>"opts"})

This outputs a file ensemble_1_opts.txt, which has two sections, background and classes. Background has two subsections include and exclude. Sections and subsections are identified by the enclosing begin <section_name> and end <section_name> statements. We provide the automatically generated template file with this document.

The include section has a list of atoms and rules that are to be *included* in the background. The exclude section has a similar list of elements that are to be *excluded* from the background. The default heuristic makes the following choices:

1. All free binding sites are included in background. All bonds are excluded.
2. When internal states are encountered on a component, the first such internal state is included in the background. This typically excludes default states such as unphosphorylated state.
3. All unidirectional rules are excluded from background.
4. For bidirectional rules, the forward rule is excluded from background, but the reverse rule is included.
5. All molecule atoms, if any, are excluded from background.

In the classes section, patterns that have been excluded from the background are sorted into a number of classes. Each class is denoted by begin <classname> and end <classname> statements enclosing a list of atoms. The default heuristic for grouping makes the following choices:

1. Bond atoms are grouped based on the molecules they link, e.g., Ste11(ste5!1).Ste5(ste11!1) is placed in the group Ste11|Ste5.
2. Internal state atoms are grouped based on the molecule they are present in and the states they represent, e.g., Ste7(S359_T363~p) is placed in the group Ste7_p.

# S2.6 User modifications to Default Template

To incorporate model-specific functional prioritization and annotation, the provided template file may be modified manually.

- Atoms and rules may be moved between include and exclude sections
- New classes of atoms may be added to the classes section
- Existing classes may be removed.

We use ‘move to background’ to denote atoms or rules moved from exclude to include sections and ‘move to foreground’ to denote the converse.

In the Suderman and Deeds model, all dissociation rules were modeled as separate rules. We identify rules that are unproductive reverses of binding rules and move them to background. However, a subset of dissociation rules are not considered unproductive, e.g., dissociation of Gpa1(ste4!1).Ste4(gpa!1), which leads to formation of the signaling complexes with Ste5. These rules are retained in the foreground. Other rules moved to background include dephosphorylation and molecule synthesis/deletion rules that are not initiated by the pheromone signal. Among atoms, all molecule atoms are moved to background. Among the free binding site patterns, those whose freely-diffusing state can be considered important were moved to foreground, e.g., Pheromone(ste2). Internal state patterns for which all alternate states were considered important were moved to foreground, e.g., Ste5(loc~n)/Ste5(loc~c) and Gpa1(nuc~GDP)/Gpa1(nuc~GTP). The Suderman and Deeds model is fairly simple in terms of structural composition, so most groups have only member each.

The modifications to the classes section include correcting the choices of the default heuristic as well as adding new classes for those atoms that were moved to foreground. For the Suderman and Deeds model, the class Ste11_p comprised of Ste11(S302_S306_S307~p) and Ste11(degradation~p) was split into two classes named Ste11_p and Ste11_deg respectively. Free binding sites were grouped by molecule, e.g., Ste12(dna) would be placed in the group Ste12_free. Other classes were added for internal state patterns moved to foreground such as Ste5_n for Ste5(loc~n) and Gpa1_GDP for Gpa1(nuc~GDP). The modified template file is provided with this document as opts.txt, with the modifications made indicated by inline comments.

# S2.7: Compressed AR Graph

To generate the compressed AR graph, run ensemble_1.bngl with the action:

visualize({type=>"regulatory",opts=>"opts.txt",groups=>1,collapse=>1,doNotUseContextWhenGrouping=>1,ruleNames=>1,removeReactantContext=>1,suffix=>"compressed"})

The opts.txt used here refers to the modified template file generated in the previous step. Since background=>1 is not mentioned, background nodes are not shown. Flags groups=>1 and collapse=>1 call the grouping and collapsing methods of complexity reduction respectively. The uncompressed version of the graph with group nodes added can be accessed using collapse=>0. The flag doNotUseContextWhenGrouping=>1 indicates that the permissive edge signature for grouping is to be used (strict is default). The flag removeReactantContext=>1 is to be used when groups representing freely-diffusing states have been added manually, as is the case for this model. The output of this command is the file ensemble_1_regulatory_compressed.gml. When imported into yEd, it looks like this:


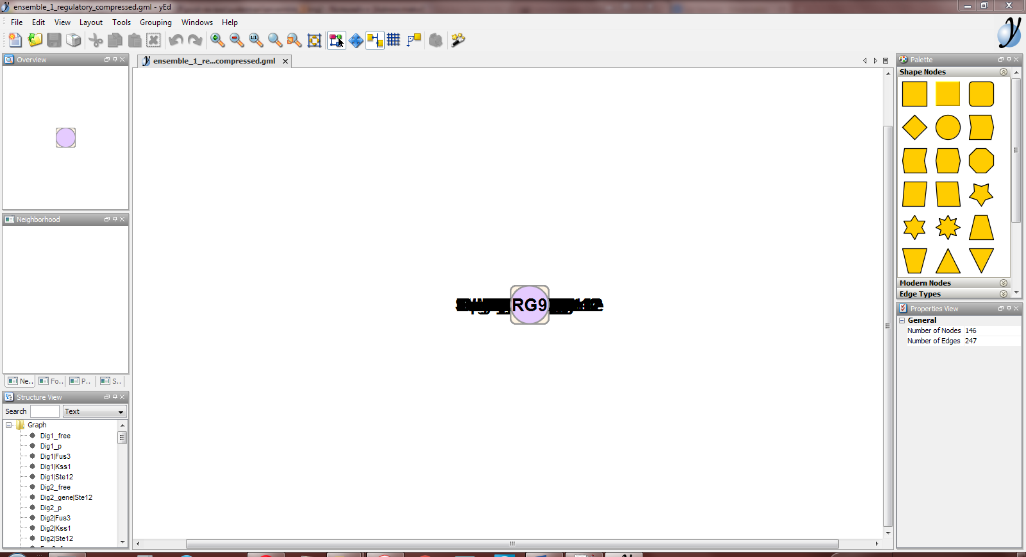


Rule groups are named RG0, RG1, etc. Rules that did not share an edge signature with any other rule retain their names. Select the node Pheromone from the Structure View panel on the bottom left. Then select Layout->Radial from the menu. In the dialog that pops up, set Center Allocation Policy as “Selected Nodes”, Maximal Child Sector Size as 270, and Routing Style as “Straight”, then click Ok. The diagram is now laid out like this:


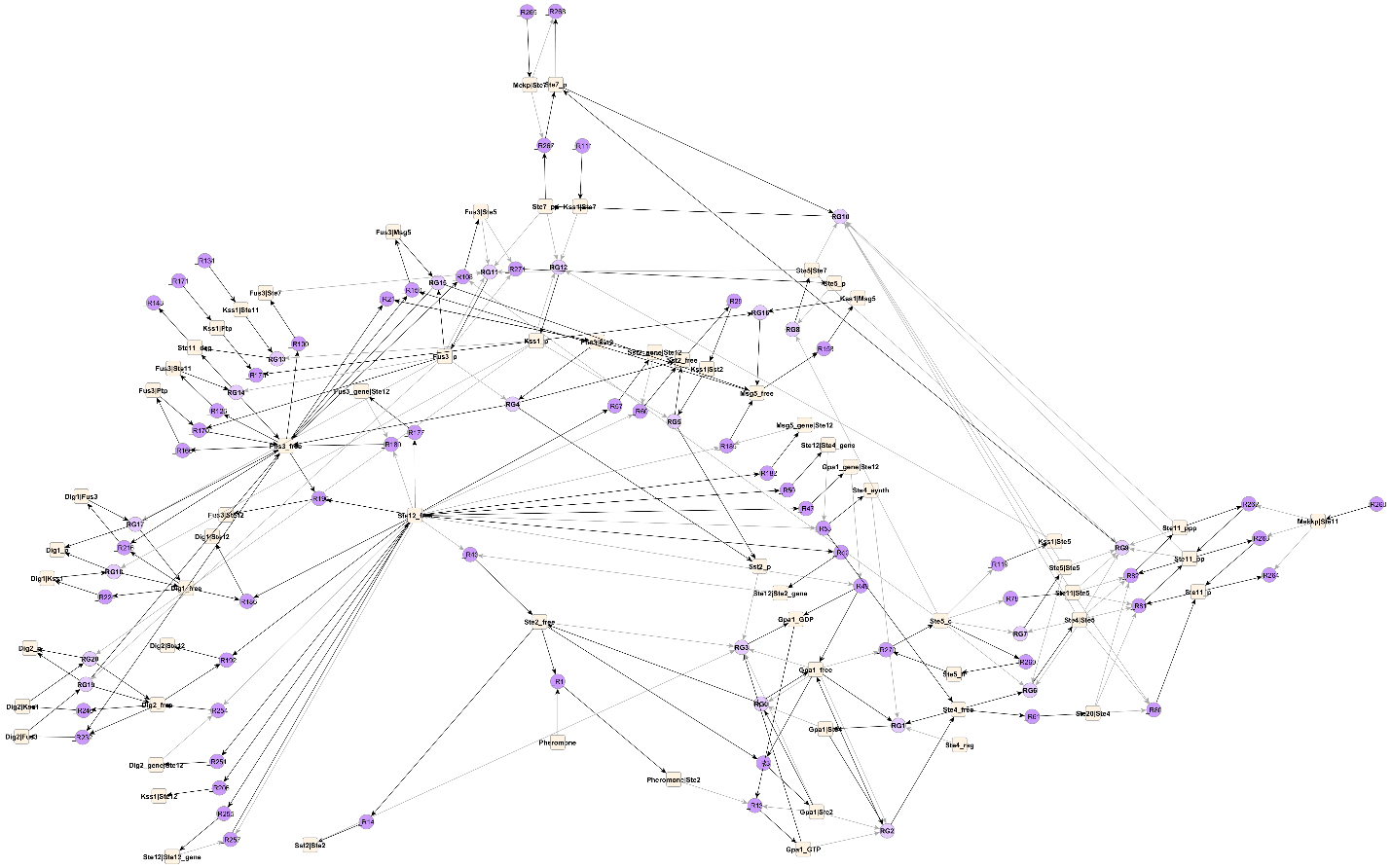


The laid out file ensemble_1_regulatory_compressed.gml is provided as attachment. Note the Pheromone node at the center, from which signal is initiated in the model. Other important ‘hub’ nodes in the network include Ste2_free, Ste5_c, Ste12_free and Fus3_free.

Subsets of the graph can be explored by creating selections. For example, select Ste12_free on this map, then select Tools->Select Elements from the menu. A dialog box opens up with multiple tabs. In the General tab, uncheck “Clear Selection First”. In the Nodes tab, check the selection box for “Use these criteria”, select “Neighbors of Selected Nodes” from the Select drop-down menu, and set Maximal Path Distance as 2, then click Ok. The resulting selection on the map consists of Ste12_free and all nodes that are at most 2 edges away. From the menu, select Edit->Copy to copy these nodes, File->New to create a new file, then Edit->Paste to paste the copied nodes into the new file. Then select Tools->One-Click Layout to get the following image. We provide the new diagram as the file Ste12.gml.


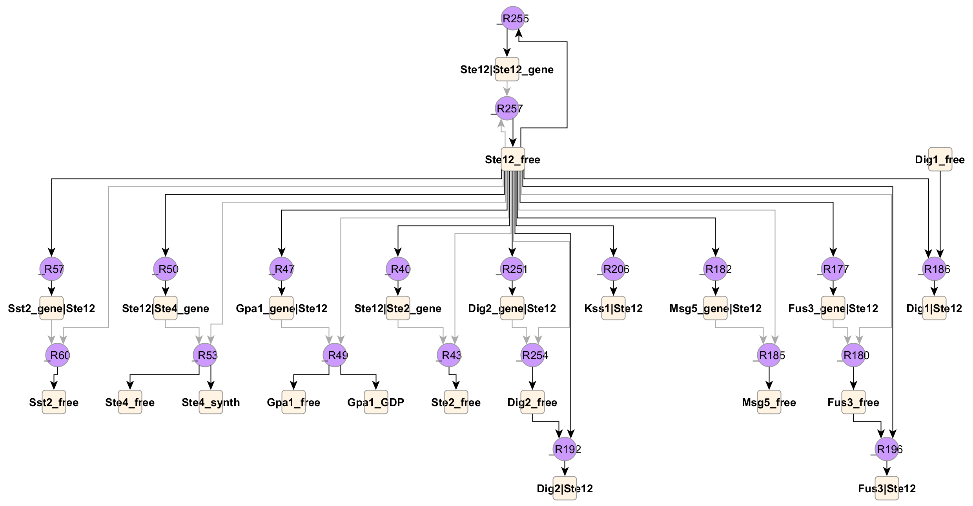


The diagram captures the functional interactions of Ste12_free, including:

- A positive feedback loop between Ste12 and its gene.
- Transcription factor activity of Ste12 on Sst2, Ste4, Gpa1, Ste2, Dig2, Msg5 and Fus3 genes.
- Competitive binding interactions with Dig1, Dig2, Fus3 and Kss1.

Note that a rule may have complex contexts, requiring a number of different states and binding interactions. Every atom that can be identified in these contexts will be represented on the AR graph, e.g., the subset of the graph showing _R82 is given by


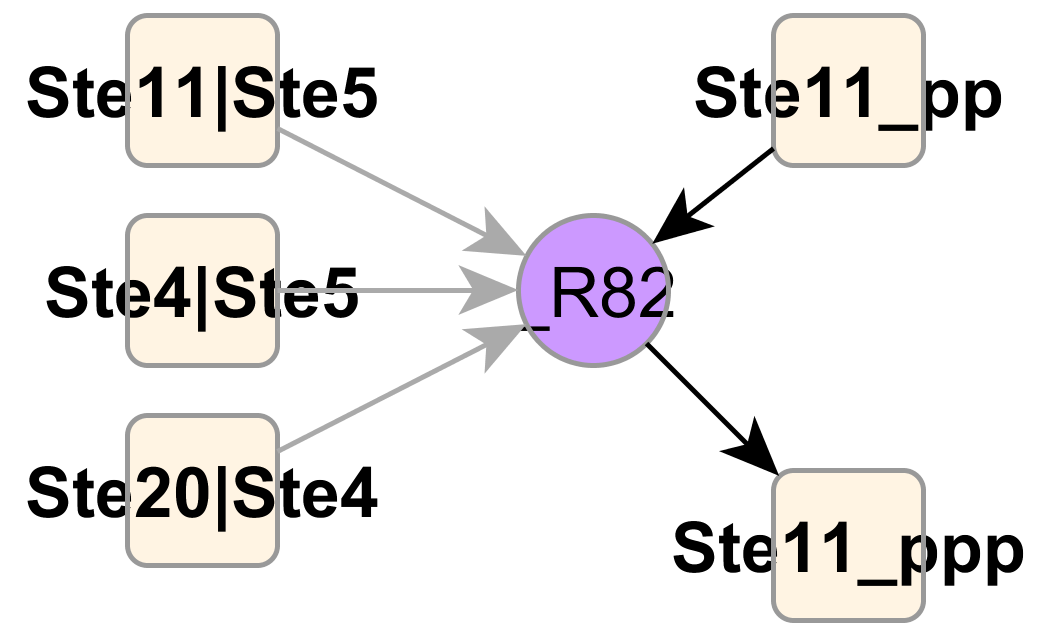


Here, the rule phosphorylates Ste11_pp to Ste11_ppp, and it requires the binding interactions Ste11|Ste5, Ste4|Ste5 and Ste20|Ste4.
