# Supplementary material for: Automated visualization of rule-based models
Source: PLoS Comput Biol. 2017 Nov 13;13(11):e1005857. doi: 10.1371/journal.pcbi.1005857 (PMC5703574; doi:10.1371/journal.pcbi.1005857)
Supplement: S1 Table — (DOCX) [file pcbi.1005857.s008.docx]

S1 Table: List of Rule-based Models

| **No.** | **Citation** | **System** | **Rules** |
| --- | --- | --- | --- |
| 1 | **An et al. 2009** Math BioSci. | TLR4 | 59 |
| 2 | **Barua et al. 2008** J Biol Chem. | PDGFRβ | 43 |
| 3 | **Barua et al. 2009** PLoS Comput Biol. | GH-Jak | 6 |
| 4 | **Barua et al. 2012** J Immunol. | BCR | 44 |
| 5 | **Barua et al. 2012** PLoS One | FcεRI | 86 |
| 6 | **Barua et al. 2013** PLoS Comput Biol. | APC | 79 |
| 7 | **Blinov et al. 2006** Biosystems | EGFR | 39 |
| 8 | **Chylek et al. 2014** Front Immunol. | FcεRI | 145 |
| 9 | **Chylek et al. 2014** PLoS One | TCR | 158 |
| 10 | **Creamer et al. 2012** BMC Syst Biol. | ErBB | 625 |
| 11 | **Dushek et al. 2011** Biophys J. | Multi-site phosphorylation | 128 |
| 12 | **Dushek et al. 2014** Biophys J. | FRET Biosensors | 10 |
| 13 | **Faeder et al. 2003** J Immunol. | FcεRI | 24 |
| 14 | **Falkenberg et al. 2013** Biophys J. | TLBR | 6 |
| 15 | **Hat et al. 2016** PLoS Comput Biol. | p53 | 94 |
| 16 | **Kesseler et al. 2013** J Theor Biol. | G2 checkpoint | 173 |
| 17 | **Kocieniewski et al. 2012** J Theor Biol. | MAPK | 28 |
| 18 | **Kozer et al. 2013** Mol Biosyst. | EGFR | 26 |
| 19 | **Kozer et al. 2014** Biochemistry | EGFR-Grb2 | 28 |
| 20 | **Ligon et al. 2014** PLoS One | Lipoplex transfection | 33 |
| 21 | **Michalski et al. 2012** Phys Biol. | CaMKII | 8 |
| 22 | **Mukhopadhyay et al. 2013** PLoS Comput Biol. | TCR | 42 |
| 23 | **Nag et al. 2009** Biophys J. | Lat-Grb2-SOS1 | 52 |
| 24 | **Pekalski et al. 2013** PLoS One | TNFα | 40 |
| 25 | **Stites et al. 2015** Biophys J. | EGFR | 178 |
| 26 | **Szymanska et al. 2015** PLoS One | MTORC1 | 31 |
| 27 | **Thomson et al. 2011** PNAS | Yeast GPCR | 54 |
